# Metabolic readouts of tumor instructed normal tissues (TINT) identify aggressive prostate cancer subgroups for tailored therapy

**DOI:** 10.3389/fmolb.2025.1426949

**Published:** 2025-04-07

**Authors:** Ilona Dudka, João Figueira, Pernilla Wikström, Anders Bergh, Gerhard Gröbner

**Affiliations:** ^1^ Department of Chemistry, Umeå University, Umeå, Sweden; ^2^ Department of Medical Biosciences, Pathology, Umeå University, Umeå, Sweden

**Keywords:** metabolomics, prostate cancer, TINT -tumor instructed normal tissue, HR MAS NMR, biomarker

## Abstract

**Introduction:**

Prostate cancer (PC) diagnosis relies on histopathological examination of prostate biopsies, which is restricted by insufficient sampling of all tumors present. Including samples from non-PC but tumor instructed normal tissues (TINT) may increase the diagnostic power by displaying the adaptive responses in benign tissues near tumors.

**Methods:**

Here, we applied high-resolution magic angle spinning nuclear magnetic resonance (HR MAS NMR) to identify metabolomic biomarkers of possible diagnostic value in benign prostate tissues near low/high-grade tumors.

**Results:**

Benign samples near high-grade tumors (B ISUP 3 + 4) exhibited altered metabolic profiles compared to those close to low-grade tumors (B ISUP 1 + 2). The levels of six metabolites differentiated between the two groups; myo-inositol, lysine, serine and combined signal of lysine/leucine/arginine were increased in benign samples near high-grade tumors (B ISUP 3 + 4) compared to near low-grade tumors (B ISUP 1 + 2), while levels of ethanolamine and lactate were decreased. Additionally, we revealed metabolic differences in non-cancer tissues as a function of their distance to the nearest tumor. Eight metabolites (glutathione, glutamate, combined signal of glutamate/glutamine - glx, glycerol, inosine, ethanolamine, serine and arginine) differentiated between benign tissue located close to the tumor (d ≤ 5 mm) compared to those far away (d ≥ 1 cm).

**Conclusion:**

Our HR MAS NMR-based approach identified metabolic signatures in prostate biopsies that reflect the response of benign tissues to the presence of nearby located tumors in the same prostate and confirmed the power of the TINT concept for improved PC diagnostics and understanding of tumor-tissue interactions.

## 1 Introduction

A reliable diagnosis of prostate cancer (PC) depends on a histopathological examination of biopsies (Mottet et al., 2021) usually performed upon initial determination of prostate serum antigen (PSA) levels, digital rectal exam (DRE), and guided by ultrasound and magnetic resonance imaging (MRI). However, routine biopsies, due to the low prostate volume examined, sometimes miss to sample all tumors present, and they may fail to sample the most aggressive foci (Hammarsten et al., 2010). The successful treatment of PC depends critically on the grading of all clinically significant tumor foci and the correct determination of their aggressiveness and capacity to form metastases (Teo et al., 2019). Therefore, to provide a reliable diagnostic procedure another level of potentially high information value could be included, where specific and reliable PC biomarkers in sampled non-malignant prostate tissue located near tumor foci are considered. Ideally, those nearby biomarkers should indicate not only the presence, but also the aggressiveness of tumors located elsewhere in the prostate (Hammarsten et al., 2010).

The concept named TINT - tumor instructed normal tissue, was established by Bergh and co-workers over a decade ago (Halin et al., 2010). The TINT concept is based on a tumor induced adaptive response in histologically normal-appearing epithelium and stroma. This tumor interaction with nearby benign tissues allows subsequent growth and spreading of the tumor into the surrounding tissue environment. However, the TINT does not need to be in direct contact with the cancer epithelium and should not be mixed with the tumor stroma or the tumor microenvironment (Adamo et al., 2015a). TINT is also different from the so-called field cancerization or field effect, which describes pre-malignant genotypic and phenotypic alterations required for the transformation of cancer cells (Curtius et al., 2018).

Recently, the diagnostic power of the TINT concept was demonstrated by monitoring the adaptation of morphologically benign prostate tissues to nearby located tumors using rat PC models (Adamo et al., 2015a). Additionally, in small localized PCs increased DNA synthesis was observed in remote tissue regions in a tumor type- and size-dependent manner (Halin et al., 2022). On a phenotypic level, the expression of some genes in the TINT is changed in a similar way as in tumors, while other changes seem to be exclusive to the TINT (Adamo et al., 2015b). Aggressive tumors may have particular effects on adjacent tissues and alterations in the non-malignant tissue located close to tumor sites can therefore provide valuable prognostic information. Low levels of phosphorylated epidermal growth factor receptor found in non-malignant and malignant prostate tissue predict favourable outcome for PC patients (Hammarsten et al., 2010). In contrast, high levels of lysyl oxidase (LOX) in the non-malignant prostate epithelium are correlated with poor outcomes (Nilsson et al., 2015). Additionally, the hyaluronan staining score in the surrounding morphologically normal prostate tissue is associated with tumor aggressiveness and increased mortality risk (Josefsson et al., 2011). Similar correlations were found for microseminoprotein-beta (MSMB) and C/EBPβ expression levels found in surrounding tissue areas (Bergstrom et al., 2018; Adamo et al., 2018). Moreover, in the stroma of tumors and in non-malignant prostate tissues, a high number of S100A9 positive inflammatory cells is associated with shorter cancer-specific survival (Tidehag et al., 2014), and a low stroma androgen receptor level is related to poor outcomes in PC patients (Wikstrom et al., 2009).

In recent years, metabolomic profiling has also contributed to a more comprehensive molecular understanding of PC, including understanding of the intracellular signalling pathways regulating prostate carcinogenesis (Rye et al., 2022; Andersen et al., 2021; Bruzzone et al., 2020). Metabolomic analysis has provided specific biomarkers for aggressive PC subtypes, which may be useful for precision medicine (Hansen et al., 2023; Dudka et al., 2020). Here, we further expand the potential of the TINT concept for diagnostics and therapy by identifying metabolomic biomarkers with potentially high prognostic value in the clinic. For this purpose, we used the high resolution magic angle spinning nuclear magnetic resonance (HR MAS NMR) technique on intact prostate biopsies to provide metabolic signatures for two sets of benign prostate samples characterized by surrounding PC tumor tissue of low (B ISUP 1 + 2) and high (B ISUP 3 + 4) ISUP grades. In this way we identified six metabolites in non-cancer tissues that changed significantly as a function of the tumor grade in the surrounding tissues. Additionally, we observed that changes in eight metabolites in benign samples were related to distances to the closest tumor foci. Our findings indicate the possibility of exploiting molecular markers for reliable early diagnosis and prognosis, even in tumor-negative biopsies, which is often the case in routine clinical settings. We identified biomarkers that can differentiate between non-cancer tissues located near high-grade *versus* low-grade PC. Therefore, this portfolio of “TINT” biomarkers provides a powerful diagnostic tool for identifying patients with negative biopsies who are in high need of close follow-up and possibly early diagnosis and treatment.

## 2 Materials and methods

### 2.1 Patients and tissue samples

In this study we used a well-characterized patient cohort as described previously (Dudka et al., 2023). The study was conducted in accordance with the Declaration of Helsinki, and the study protocol was approved by the research ethical committee at Umeå University Hospital (Regional Ethical Review Board in Umeå). Written informed consent was obtained from each patient. Biopsy prostate samples were obtained from 31 patients treated by prostatectomy at the Urology Clinic, Umeå University Hospital, between 2009–2012. Patient ages ranged between 58 and 74 years and preoperative serum PSA levels between 2.8 and 28 μg/L. No prostate cancer treatment had been given prior to surgery. A detailed description of sample collection is described in (20). Shortly, immediately after surgical removal the prostates were brought to the Pathology Department and cut in 0.5 cm thick slices. From each prostate 20 samples were punched from the slides using a 0.5 cm steel cylinder and frozen in – 70°C within 30 min after surgery. The prostate slices were then fixed in 4% formaldehyde for 24 h, dehydrated, embedded in paraffin (FFPE), cut in 5 µm thick sections and stained with hematoxylin–eosin (H&E). The different frozen samples could thus be identified as individual holes in the paraffin sections. From the metabolomics profiles of 104 samples (48 benign and 56 cancer) analysed in the previous study, we selected 57 samples (27 benign and 30 cancer) from patients with unifocal cancer and additionally 35 samples (17 benign and 18 cancer) from patients with multifocal cancer, with the following conditions fulfilled: the tumor is the index tumor (highest grade and largest), the normal sample is taken from the same side, and the other tumors are located on the contralateral side and at even larger distances. The Gleason grade and ISUP grade were estimated for the cancerous tissue samples. For benign samples, Gleason grade and ISUP grade were determined based on the assignment of the closest tumor. Additionally, benign samples were characterized by distance to the closest tumor. The clinicopathological characteristics of the patients and samples are summarized in Table 1. Because of observed heterogeneity of benign/tumor samples from the same patient each replicate was treated as an individual sample in the metabolic analysis.

**TABLE 1:** Patient and sample characteristics.

	ISUP grade	Unifocal and selected multifocal samples	d ≤ 5 mm	5 mm < d ≤ 1 cm	1 cm < d ≤ 1.5 cm	1.5 cm < d ≤ 2 cm	2 cm < d ≤ 2.5 cm	2.5 cm < d ≤ 3 cm	Unifocal samples
Patients		28							19
Malignant samples		48							30
ISUP grade group /Gleason score of tumor	1/3 + 3	3							0
	2/3 + 4	28							15
	3/4 + 3	13							11
	4/4 + 4	4							4
Benign samples		(44)* 40	18	9	7	2	2	2	(27)* 24
ISUP grade group /Gleason score of the closest tumor	1/3 + 3	(4)* 3	1	2	0	0	0	0	(0)* 0
	2/3 + 4	(32)* 29	13	7	5	1	1	2	(20)* 17
	3/4 + 3	(6)* 6	3	0	2	1	0	0	(5)* 5
	4/4 + 4	(2)* 2	1	0	0	0	1	0	(2)* 2

*Numbers in brackets indicate number of benign samples including four samples that did not have accompanying tumor sample, so it was not possible to assign distance to the closest tumor.

### 2.2 ^1^H HR MAS MRS experiments

Metabolomic analysis of prostate tissue samples was carried out as described previously (Dudka et al., 2023). Briefly, NMR experiments were carried out on a 500 MHz NMR spectrometer (Bruker Biospin, GmbH, Germany) at 277 K. We used a Carr-Purcell-Meiboom-Gill (CPMG) NMR pulse sequence with a spectral width of 20 ppm, 1,024 scans, echo time of 0.2 m, a total acquisition time of 1.64 s, a recycle delay of 1.5 s and 32 K data points. The acquired spectral data were baseline and phase corrected using TopSpin 3.6.5 software (Bruker Biospin, GmbH, Germany). Prior to peak integration seven spectra were excluded due to high levels of lipids as indicated by their pronounced NMR signals in those samples. The NMR spectra were aligned using icoshift 1.2 MATLAB 2017a (The Mathworks, Inc., United States). Integration of peaks was performed to a linear baseline on all spectra in parallel to overcome broad signals in the spectra using an in-house developed MATLAB R2017a routine. Thus, the relative abundances of metabolites in the study groups were obtained and subjected to further analysis as used previously in our studies (Dudka et al., 2020; Dudka et al., 2023). Metabolite identification was carried out using the Chenomx NMR suite professional (version 8.6, Chenomx Inc., Edmonton, Canada). Only those metabolites that, according to the literature, have a large contribution to a certain integrated peak region were assigned. In overlapping peak regions of the spectra few metabolites were assigned to one integrated peak.

### 2.3 Multivariate analysis

Multivariate analysis of the NMR spectra was carried out in SIMCA V17 (Umetrics, Umeå, Sweden). Principal component analysis (PCA) was performed on the spectral data set to evaluate the homogeneity of the samples and identify any possible trends and outliers between the samples. Thereafter, supervised multivariate analysis orthogonal partial least squares discriminant analysis (OPLS-DA) was performed to visualize the differences between assigned groups by reducing the high dimensionality into predictive and orthogonal latent variables. Analysis of variance of cross-validated predictive residuals (CV-ANOVA) was used to assess the significance of the OPLS-DA models, where a *p* value lower than 0.05 was associated with a significant model. Leave-one-out cross validation was applied to all OPLS-DA models. Additionally, permutational multivariate analysis of variance (PERMANOVA) analysis was performed to evaluate the differences in tissue metabolomes between assigned groups and individual patients. The PERMANOVAs were performed using the pairwiseAdonis package of R software.

### 2.4 Univariate statistical analysis

Metabolomic differences among assigned groups were tested by using the t-test (for data which did not significantly deviate from normality) or Mann-Whitney test (for data which significantly deviated from normality) for two-groups comparisons and one-way ANOVA followed by Tukey’s multiple comparisons test if data was normally distributed, otherwise Kruskal–Wallis test followed by Dunn’s test for three or more group comparisons. Additionally, Benjamini–Hochberg correction for multiple comparisons (q < 0.05) was applied. *Pp*Correlations between metabolite levels and ISUP values and distance (d) from the tumor values were calculated with Pearson correlation (two-tailed *p* value, 95% confidence interval). Univariate statistical analyses were performed with GraphPad Prism (GraphPad Software Inc., San Diego, CA, United States) version 9.4.1.

## 3 Results

### 3.1 Study description and sample characteristics

First, we aimed to identify metabolomic changes in benign prostate biopsies from PC patients associated with their location near malignant tissue of varying aggressiveness. Second, we wanted to explore variations in metabolic levels in those benign samples as a function of their distance from malignant tissue. To obtain highly reliable findings we used a well-characterized study cohort and previously obtained tissue metabolomics profiles (Dudka et al., 2023). With the application of HR MAS NMR, 92 variables were integrated, from which 48 metabolites could be identified and quantified in the main NMR spectral region (0.7–8.5 ^1^H ppm) as presented in Supplementary Table S1. A representative 500 MHz ^1^H HR MAS NMR spectrum of individual prostate tissue sample is shown in Supplementary Figure S1. In our analysis, we included only samples from patients with unifocal cancer and from patients with multifocal cancer where we assumed that changes in the benign samples were due to the indexed tumor and not an effect of other tumor foci.

Finally, 92 samples (44 benign and 48 cancer) were used for multivariate and univariate analyses. Each benign sample had additional information regarding the distance to the closest tumor and the corresponding tumor ISUP grade, with the exception for four samples where distance to the closest tumor was not known. Benign samples were divided into six groups according to the distance (d) to the tumor: group 1 (d ≤ 5 mm, n = 18), group 2 (5 mm < d ≤ 1 cm, n = 9), group 3 (1 cm < d ≤ 1.5 cm, n = 7), group 4 (1.5 cm < d ≤ 2 cm, n = 2), group 5 (2 cm < d ≤ 2.5 cm, n = 2), and group 6 (2.5 cm < d ≤ 3 cm, n = 2). In the context of ISUP grade assignment, 4 benign samples belonged to ISUP 1, 32 to ISUP 2, 6 to ISUP 3 and 2 samples to ISUP 4. Due to the limited number of samples, especially for ISUP 1 and 4, benign samples with ISUP 1 and 2 were combined into one group (B ISUP 1 + 2, n = 36) reflecting benign samples adjacent to less-aggressive tumors, and ISUP 3 and 4 were combined into a second group (B ISUP 3 + 4, n = 8) containing benign samples adjacent to more aggressive tumors.

Comparisons between cancer and benign samples and between different PC subtypes were presented in a previous paper (Dudka et al., 2023). From the group of benign samples, two patients had two samples from the same biopsy included in analysis. Other samples from the same patients are benign samples accompanying different tumor biopsies/tumor foci of that individual. From the group of tumor samples, seven patients had two samples included from the same biopsy and two had three samples. Table 1 summarizes the histological characteristics of the samples used in our final analysis. Results of PERMANOVA analysis showed that the stratification of benign and malignant samples from patients with unifocal and multifocal cancer analysed together (*p* = 0.001) and from unifocal samples only (*p* = 0.001) were significant (Supplementary Figure S2A), indicating that the number of samples per patients were not a significant factor for this group separation.

### 3.2 Benign prostate tissues classified based on ISUP grades of accompanying tumors

**FIGURE 1:**
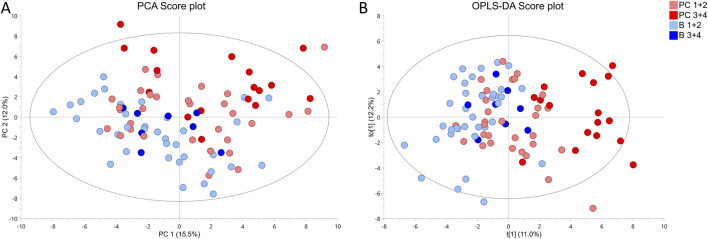
^1^ ^1^H HR MAS NMR profiling of benign (B ISUP 1 + 2 and B ISUP 3 + 4) and malignant (PC ISUP 1 + 2 and PC ISUP 3 + 4) tumor tissue samples from prostate cancer (PC) patients with unifocal and selected multifocal tumors. **(A)** PCA score plot. Results of PERMONOVA analysis: PC 1 + 2 vs*.* PC 3 + 4 *p*-value = 0.018, PC 1 + 2 vs*.* B 1 + 2 *p*-value = 0.09, PC 1 + 2 vs*.* B 3 + 4 *p*-value = 0.726, PC 3 + 4 vs*.* B 1 + 2 *p*-value = 0.006, PC 3 + 4 vs*.* B 3 + 4 *p*-value = 0.018, B 1 + 2 vs*.* B 3 + 4 *p*-value = 1. **(B)** OPLS-DA score plot, where R2Y = 0.224 and Q2 = 0.150, *p* = 1.97 × 10^−8^ from CV-ANOVA. Number of cross-validation groups was equal the number of samples (n = 92). The model was characterized by one predictive and one orthogonal component (1 + 1).

To understand how cancer tissue of different grades affects the metabolic fingerprint of surrounding benign tissue, collected benign samples were investigated by HR MAS NMR. The metabolic profiles of benign prostate tissue samples were modelled according to their proximity to cancer of either ISUP 1 + 2 or 3 + 4. In PCA and OPLS-DA (R2Y = 0.224 and Q2 = 0.150, *p* = 1.97 × 10^−8^ from CV-ANOVA, one predictive and one orthogonal component (1 + 1), 92 cross-validation groups) models constructed based on samples from patients with unifocal and multifocal cancer analysed together, *p* ^−8^ we compared two benign groups (B ISUP 1 + 2 and B ISUP 3 + 4) and two cancer groups (PC ISUP 1 + 2 and PC ISUP 3 + 4) as presented in Figure 1. Here, tumor samples from the ISUP 3 + 4 group were generally separated from the other samples, while clear overlaps were observed between samples from other groups. This clustering was confirmed by results of PERMANOVA analysis: PC 1 + 2 vs*.* PC 3 + 4, *p*-value = 0.018; PC 1 + 2 vs*.* B 1 + 2, *p*-value = 0.09; PC 1 + 2 vs*.* B 3 + 4, *p*-value = 0.726; PC 3 + 4 vs*.* B 1 + 2, *p*-value = 0.006; PC 3 + 4 vs*.* B 3 + 4, *p*-value = 0.018; B 1 + 2 vs*.* B 3 + 4, *p*-value = 1. When we compared all four groups using only unifocal samples, the clustering was even more clear as presented in Supplementary Figure S3A (R2Y = 0.542 and Q2 = 0.201, *p* = 0.017 from CV-ANOVA, three predictive components and one orthogonal component (3 + 1) and 57 cross-validation groups). Results of PERMANOVA analysis for this set was similar as including all samples: PC 1 + 2 vs*.* PC 3 + 4, *p*-value = 0.126; PC 1 + 2 vs*.* B 1 + 2, *p*-value = 0.048; PC 1 + 2 vs*.* B 3 + 4, *p*-value = 0.100; PC 3 + 4 vs*.* B 1 + 2, *p*-value = 0.006; PC 3 + 4 vs*.* B 3 + 4, *p*-value = 0.006; B 1 + 2 vs*.* B 3 + 4, *p*-value = 1. odels comparing only the two benign groups (B ISUP 1 + 2 vs*.* B ISUP 3 + 4), including benign samples either from patients with unifocal and multifocal cancer analysed together (R2Y = 0.481 and Q2 = 0.044, *p* = 0.8401 from CV-ANOVA, one predictive component, 44 cross-validation groups) or from patients with unifocal tumors (R2Y = 0.618 and Q2 = 0.017, *p* = 0.818 from CV-ANOVA, one predictive component, 27 cross-validation groups), were not significant, probably due to the limited number of benign samples with ISUP 3 + 4 (score plots not shown).

**FIGURE 2:**
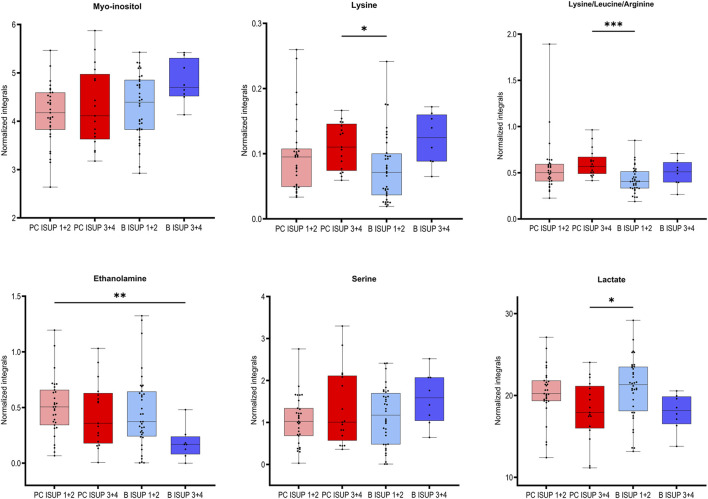
Box plots for the selected metabolites differentiating between two groups of benign prostate tissues (B ISUP 1 + 2 and B ISUP 3 + 4) and malignant tissue (PC ISUP 1 + 2 and PC ISUP 3 + 4). ANOVA followed by Tukey’s multiple comparisons test if data was normally distributed, otherwise Kruskal–Wallis test followed by Dunn’s test. Analysis was based on samples from PC patients with unifocal and selected multifocal tumors. *P < 0.05.

We selected six identified metabolites (myo-inositol, lysine, ethanolamine, serine and lactate and combined signal of lysine/leucine/arginine) that were significantly correlated with the ISUP grades of the associated tumors as presented in Table 2. Based on univariate statistical analysis (t-test/Mann-Whitney test) four of those six metabolites had significantly different levels between benign samples from the ISUP 1 + 2 and ISUP 3 + 4 groups (Table 2). However, none of those metabolites was significant after applying Benjamini–Hochberg correction for both analyses. In addition, for those six metabolites one-way ANOVA (*p* values <0.05) followed by Tukey’s multiple comparisons test or Kruskal–Wallis test followed by Dunn’s test was performed for four groups, two benign and two tumor (B ISUP 1 + 2, B ISUP 3 + 4, PC ISUP 1 + 2, PC ISUP 3 + 4). The results of this analysis are presented in Table 2 and in Figure 2. As shown in Figure 2, the levels of all metabolites of benign ISUP 3 + 4 samples in comparison to those of the benign ISUP 1 + 2 group mirrored the pattern observed for tumor tissues of the ISUP 3 + 4 grade *versus* ISUP 1 + 2 tumors. Moreover, after plotting those metabolites separately for each benign ISUP group (Supplementary Figure S4A), increasing levels of myo-inositol, lysine and a peak for lysine/leucine/arginine were observed from benign samples with ISUP 1 through benign ISUP 2, ISUP 3 and, ultimately, ISUP four displaying the highest levels. The opposite trend was observed for lactate, while the level of ethanolamine and serine did not follow a clear trend. Results of statistical analysis for all integrated variables are presented in Supplementary Table S2 (for samples from patients with unifocal and selected multifocal tumors) and S3 (for samples from patients with only unifocal tumors). Additionally, the same analysis is provided for comparison of PC 1 + 2 and PC 3 + 4 for samples from patients with unifocal and selected multifocal tumors as presented in Supplementary Table S4 and unifocal samples only in Supplementary Table S5.

**TABLE 2:** Metabolites significantly changed between benign samples accompanying tumors with ISUP 1 + 2 or ISUP 3 + 4 from patients with unifocal and selected multifocal tumors (n = 44).

Metabolite	Correlation with ISUP values		B ISUP 1 + 2 vs. B ISUP 3 + 4	One-way anova PC ISUP 1 + 2 vs. PC ISUP 3 + 4 vs. B ISUP 1 + 2 vs. B ISUP 3 + 4	Post-hoc analysis B ISUP 1 + 2 vs. B ISUP 3 + 4
	Coefficient	*p*-value*	*p*-value*	p-value	*p*-value*
Myo-inositol	0.393	0.008/0.152	0.047^a^/0.395	0.1000	0.217/0.999
Lysine	0.428	0.004/0.113	0.023^b^/0.367	0.008	0.063/0.999
Lysine/Leucine/Arginine	0.309	0.041/0.317	0.166^b^/0.668	0.001	0.999/0.999
Ethanolamine	−0.367	0.014/0.152	0.013^b^/0.367	0.019	0.064/0.999
Serine	0.351	0.020/0.164	0.110^a^/0.639	0.200	0.387/0.999
Lactate	−0.360	0.017/0.152	0.037^a^/0.367	0.018	0.126/0.999

*For the second value per column Benjamini–Hochberg correction was applied.

^a^ t-test.

^b^ Mann-Whitney test.

### 3.3 Classification of benign prostate tissue according to the distance to the nearest tumor

**FIGURE 3:**
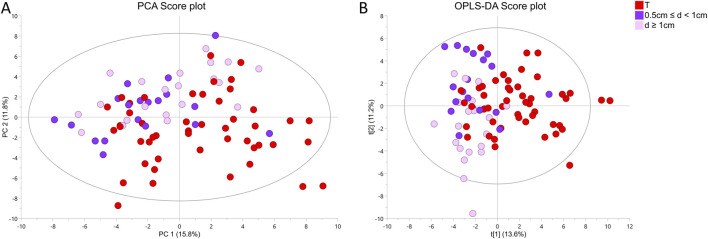
^1^H HR MAS NMR profiling of two groups of benign prostate samples based on tissue distances (d) to cancer foci (group 1: 0.5 cm ≤ d < 1 cm and group 2: d ≥ 1 cm) and one group of tumor samples (T) from prostate cancer (PC) patients with unifocal and selected multifocal tumors **(A)** PCA score plot of two benign groups with different distances to the closest cancer foci and one group with tumor samples. Results of PERMONOVA analysis: PC vs*.* 0.5 cm ≤ d < 1 cm *p*-value = 0.003, PC vs*.* d ≥ 1 cm *p*-value = 0.003, 0.5 cm ≤ d < 1 cm vs*.* d ≥ 1 cm *p*-value = 0.138. **(B)** OPLS-DA score plot corresponding to the same samples as in A, where R2Y = 0.304 and Q2Y = 0.161, *p* = 1.62 × 10^−4^ from CV-ANOVA. Number of cross-validation groups was equal the number of samples (n = 88). The model was characterized by two predictive components (2 + 0).

**FIGURE 4:**
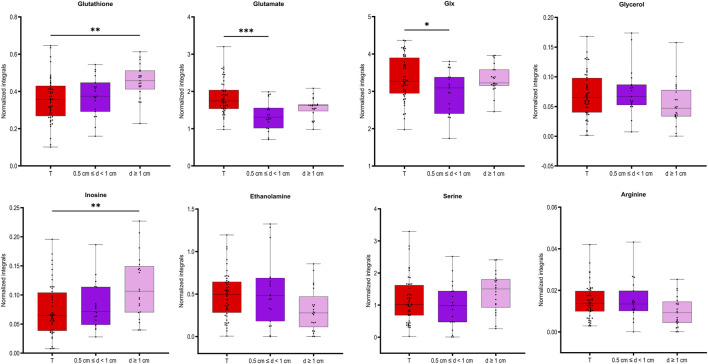
Box plots for the selected metabolites differentiating between the two groups of benign prostate samples, close to (0.5 cm ≤ d < 1) or far away from the closest tumor (d ≥ 1 cm) presented also in tumor samples. ANOVA followed by Tukey’s multiple comparisons test if data was normally distributed, otherwise Kruskal–Wallis test followed by Dunn’s test. Analysis was based on samples from PC patients with unifocal and selected multifocal tumors. *P < 0.05.

To understand how distance to cancer affects the metabolic fingerprint of benign samples, adequate benign samples were investigated by HR MAS NMR. The metabolic profiles of benign prostate tissue samples were modelled according to their distance to the closest cancer foci, with tumor tissue samples also included in the model. In supplementary Figure S3B the PCA and OPLS-DA score plots (R2Y = 0.128 and Q2Y = 0.043, *p* = 1 from CV-ANOVA, two predictive components, 88 cross-validation groups) (for samples from patients with unifocal and multifocal cancer analysed together), are presented for six distance groups of benign samples together with the tumor-containing group. Here, samples from group 1 (d ≤ 5 mm) are overlapped with a fraction of tumor samples, and samples with distance d = 1.5 cm were best separated from tumor specimens. This was underlined by PERMANOVA analysis, where the only significant result was for T vs*.* 3 cm, *p*-value = 0.042. The same analysis including only unifocal samples is presented in Supplementary Figure S3C, where the only significant result from PERMANOVA analysis was for T vs*.* 1.5 cm, *p*-value = 0.021 and OPLS-DA model was characterized by R2Y = 0.156 and Q2Y = 0.056, *p* = 1 from CV-ANOVA, two predictive components, 54 cross-validation groups. To enable a more in-depth analysis, benign samples were divided into two classification groups due to the small sample numbers. This strategy allowed a comparison of samples very close to the tumor (0.5 cm ≤ d < 1 cm) with samples far away from the tumor (d ≥ 1 cm) and with tumor samples. In this way, a PCA score plot and reliable OPLS-DA model were obtained for samples from patients with both unifocal and multifocal cancer (R2Y = 0.304 and Q2Y = 0.161, *p* = 1.62 × 10^−4^ from CV-ANOVA, two predictive components, 88 cross-validation groups) as shown in Figures 3A, B and in Fig. S3D for solely unifocal samples (R2Y = 0.375 and Q2Ycum = 0.202, *p* = 1.27 × 10^−2^ from CV-ANOVA, two predictive components, 54 cross-validation groups). Nevertheless, a few tumor samples still partially overlapped with the benign samples, especially those from group 1. Observed clustering was confirmed by results of PERMANOVA analysis for samples from patients with both unifocal and multifocal cancer: PC vs*.* 0.5 cm ≤ d < 1 cm *p*-value = 0.003; PC vs*.* d ≥ 1 cm *p*-value = 0.003; 0.5 cm ≤ d < 1 cm vs*.* d ≥ 1 cm *p*-value = 0.138; and only unifocal samples: PC vs*.* 0.5 cm ≤ d < 1 cm *p*-value = 0.042; PC vs*.* d ≥ 1 cm *p*-value = 0.003; 0.5 cm ≤ d < 1 cm vs*.* d ≥ 1 cm *p*-value = 0.189. Models based on the two benign groups with different distances to the tumor (0.5 cm ≤ d < 1 cm vs*.* d ≥ 1 cm) including either only unifocal or unifocal with multifocal samples, were not significant (plots not shown). Nevertheless, based on univariate analysis (results of t-test/Mann-Whitney test and a Pearson correlation analysis between the assigned distance from the tumor and the levels of metabolites), key metabolites could be identified to differentiate between benign samples near a tumor (0.5 cm ≤ d < 1 cm) and samples further away (d ≥ 1 cm) as summarized in Table 3. However, none of those metabolites was significant after applying Benjamini–Hochberg correction. One-way ANOVA followed by Tukey’s multiple comparisons test or Kruskal–Wallis test followed by Dunn’s test was used to compare the two benign groups with the third group, which included tumor samples. The results of this analysis are presented in Table 3 and Figure 4. The box plots in Figure 4 display the obtained levels of selected metabolites found in tumors and in the two groups of benign samples. As shown in Supplementary Figure S4B, we also plotted the levels of those metabolites for benign samples separated into three subgroups (the first group with d = 0.5–1 cm; the second group with d = 1.5–2 cm; and the third group with d = 2.5–3 cm). Here, one can clearly explore the distance-dependent metabolic signatures of benign samples. An interesting behaviour was observed for glutathione and arginine. Glutathione levels increase with the distance from the tumor, with the lowest levels in tumor samples and the highest in benign samples 2.5–3.0 cm from the tumor. For arginine, the opposite trend was observed. Results of statistical analysis for all integrated variables are presented in Supplementary Table S6 (for samples from patients with unifocal and selected multifocal tumors) and S7 (for samples from patients with only unifocal tumors).

**TABLE 3:** Metabolites significantly changed with increasing distance from the closest tumor from patients with unifocal and selected multifocal tumors (n = 44).

Metabolite	Correlation with distance to the closest tumor		d ≤ 5 mm vs. d ≥ 1 cm	One -way anova tumor vs. d ≤ 5 mm vs. 1 cm ≤ d ≤ 3 cm	Post-hoc analysis 5 mm vs. d ≥ 1 cm
	Coefficient	*p*-value*	*p*-value*	p-value	*p*-value*
Glutathione	0.343	0.030/0.573	0.013^a^/0.324	0.004	0.063/0.999
Glutamate	0.198	0.220/0.832	0.042^b^/0.324	0.0003	0.272/0.999
Glx	0.198	0.220/0.832	0.027^a^/0.324	0.041	0.109/0.999
Inosine	0.268	0.095/0.573	0.040^b^/0.324	0.005	0.171/0.999
Ethanolamine	−0.349	0.027/0.573	0.078^b^/0.397	0.011	0.171/0.999
Serine	0.258	0.109/0.588	0.042^b^/0.324	0.085	0.099/0.999
Glycerol	−0.300	0.060/0.573	0.033^b^/0.324	0.097	0.142/0.999
Arginine	−0.331	0.037/0.573	0.066^b^/0.365	0.051	0.207/0.999

d - distance to the closest tumor.

*For the second value per column Benjamini–Hochberg correction was applied.

^a^ t-test.

^b^ Mann-Whitney test.

## 4 Discussion

Here, we identified unique metabolic signatures in prostate biopsies that reflect the response of benign tissues to the presence of nearby tumors in the same prostate. By using non-destructive HR MAS NMR spectroscopy to obtain metabolic readouts of intact biopsies, subsequent histopathological characterization can be carried out and valuable metabolic information can be obtained from histologically “normal” tissues (Yakoub et al., 2010). Most importantly, the obtained metabolic profiles not only presented the impact of tumors on nearby located benign tissues but also revealed that the metabolic levels of key biomarkers were sensitive to tumor grade and distance, further supporting the TINT concept (Halin et al., 2010). We applied multivariate analysis to visualize the overall distribution of metabolite differences between the groups, as we were comparing more than two groups. The predictive values Q2 of the obtained OPLS-DA models were rather low, but we need to take into account that we are comparing relatively homogeneous classes of benign samples, so the differences between them are probably very subtle. Additionally, the significance of all models was confirmed by CV-ANOVA. Although the multivariate models obtained by analysing benign samples with respect to accompanying PC of low or high grade (B ISUP 1 + 2 vs*.* B ISUP 3 + 4) and distance to malignant tissue (0.5 cm ≤ d < 1 cm vs*.* d ≥ 1 cm) were not significant, probably due to limited number of samples, univariate comparisons identified a set of interesting metabolites that are further discussed below.

Previous metabolomics studies exploring changes in benign tissues from cancerous organs have usually interpreted them as field cancerization. Yakoub et al. (2010) observed significant and progressive changes in the levels of various metabolites in proximal histologically normal mucosa when comparing noncancer and cancer patients. Reed et al. (2017) revealed a very strong metabolic signature differentiating normal squamous epithelium from controls and esophageal adenocarcinoma patients. Using a similar NMR approach as here, Vandergrift et al. (2018) characterized the metabolomic profiles of histologically benign prostate tissues to identify tumor grade and stage and predict recurrence. In that study, elevated myo-inositol was proposed as a potential mechanistic therapeutic target in patients with highly aggressive cancer. The same group (Dinges et al., 2019) also mentioned that the metabolic signatures of benign tissues of varying distances to PC lesions can differ. To identify safe margins for rectal cancer surgery, Zhang et al. (2022) showed that the most significant changes in metabolite levels were observed at 0.5 cm (cT1 and cT2 stage) and 2.0 cm (cT3 and cT4 stage) from the tumor. An analogous concept was shown by Yang et al. (2019) in oral squamous cell carcinoma, who identified changes in amino acids as molecular markers of the surgical margin. Jimenez et al. (2013) reported that in colorectal cancer, tumor-adjacent mucosa (10 cm from the tumor margin) is characterized by unique metabolic field changes that distinguish tumors according to their T-stage and lymph node status and can accurately predict 5-year survival.

Clinically, the evaluation of PC aggressiveness is usually determined by classical histopathology of sampled prostate biopsies, including grading of observed cancer areas. The ability to detect cancer and assess aggressiveness in benign areas sampled by cancer-negative biopsies from cancer-positive patients could substantially improve the accuracy of PC diagnostics and decrease the number of unnecessary biopsies. Therefore, we focused on the concept of defining PC aggressiveness based on the metabolomic profiles of associated benign samples. The relevant OPLS-DA score plot (Figure 1B) revealed metabolomic similarities between ISUP 1 + 2 PC tumors and benign samples. Notably, all statistically significant metabolomic changes observed between benign samples from ISUP 3 + 4 and ISUP 1 + 2 groups were also noted between ISUP 3 + 4 and ISUP 1 + 2 with the levels of lactate and ethanolamine being most pronounced.

Our results clearly validate the TINT concept with the metabolomic signatures of benign prostate tissues, reflecting the aggressiveness of nearby located tumors. The differences observed in benign samples near tumors of different aggressiveness are clearly due to cancer-driven alterations in those benign tissues. Some of the identified significant metabolites in benign samples are well established and have significant malignant potential; for example, serine showing increased levels in benign ISUP 3 + 4 samples compared to benign samples with ISUP 1 + 2. This amino acid is essential for tumor growth and progression and has been suggested as oncogenesis-supportive metabolite (Geeraerts et al., 2021). In benign ISUP 3 + 4 samples, higher levels of lysine were also detected, indicating increased synthesis of collagen as described in a rat TINT model (Adamo et al., 2015b). Previous work indicated that the lysyl oxidase (LOX) score in TINT epithelium was correlated with tumor stage and PC survival (Nilsson et al., 2015). Ethanolamine was also increased in benign samples with ISUP 3 + 4 compared to those with ISUP 1 + 2. Aberrant ethanolamine phospholipid metabolism has been established as a universal metabolic hallmark of cancer (Cheng et al., 2016).

In benign tissues close to aggressive tumors, the levels of myo-inositol were also elevated. This metabolite is involved in many biological processes ranging from intracellular signal transduction, calcium homeostasis regulation to energy metabolism, and it is essential for cancer formation and metastasis (Chen et al., 2023; Case et al., 2022). Using prostate tissue extracts, inositol hexaphosphate (IP6) was shown to significantly decrease glucose metabolism and membrane phospholipid synthesis, in addition to causing an increase in myo-inositol levels in the prostate (Raina et al., 2013). Similar changes in myo-inositol in benign prostate samples were observed by Vandergrift et al. (2018). These authors suggested that myo-inositol might function as the defence response of benign tissues against nearby cancerous prostate tumors. It might thus provide endogenous tumor suppression of aggressive PC growth. Overall, elevations in the osmolyte, myo-inositol, may indicate localized changes in osmoregulation (Tian et al., 2015).

In this study, we also found metabolic changes as a function of the distance of benign samples to the next tumor locus. Here, eight metabolites were significantly different between benign tissue samples in collected close to the tumor (d ≤ 5 mm) compared to those far away from the tumor (d ≥ 1 cm). By further clustering benign samples every 5 mm from the tumor, three differently behaving benign groups could be identified. Moreover, four of those metabolites showed the largest differences between tumor and benign samples in the d = 1.5–2 cm distance region. Interestingly, level of glutathione in the benign samples gradually increased as the distance from the tumor increased. Glutathione is the primary cellular antioxidant and plays a key role in carcinogenesis and the modulation of the cellular response to antineoplastic agents (Kennedy et al., 2020). In cancer research it is important determining the cellular redox status by measurement of ratio of GSH and oxidized form of glutathione (GSSG), as molecular changes in the glutathione antioxidant system and disturbances in glutathione homeostasis are implicated in tumor initiation, progression, and treatment response. However, GSSG could not be clearly distinguished in the NMR spectra and needs further investigation. Our observation that glutathione levels increased with distance is consistent with our previous reports of downregulation of glutathione S-transferases in rat TINT models (Adamo et al., 2015b).

Dysregulation of the oxidative pathway was also visible by decreased levels of arginine and increased levels of combined signal of glutamate/glutamine (glx) and glutamate (a precursor of glutathione) in benign samples more remote from tumor locations. The observed deviation in glutamine metabolism is typical for cancer cells and drives their growth and cancer progression. PC is a tumor type that is heavily dependent on glutamine for growth and survival (Ni et al., 2023; Lasorsa et al., 2023). Interestingly, arginine showed an opposite trend relative to glutathione, with the highest levels in tumor samples and decreasing levels in benign samples as a function of their distance to the tumor. Arginine deprivation has been used to target cancers (Chen et al., 2021), and its metabolism affects not only malignant cells but also surrounding immune cells (Matos et al., 2021). Additionally, our previous studies (Dudka et al., 2020; Dudka et al., 2023) comparing benign prostate tissue samples with prostate cancer samples showed increased levels of arginine in tumor tissues. Our current results highlight that the distance of benign samples to the closest tumor can also impact this differentiation, as the lowest values were observed in benign samples further from the tumor.

Our pilot study includes unique set of benign samples and was used for biomarker discovery. However, validation of metabolomic signatures in larger and independent cohorts is still needed for further transfer to the clinics.

## 5 Conclusion

In summary, HR MAS NMR-based metabolomics of intact biopsies is shown here as a powerful approach for identifying metabolomic alterations in benign biopsies from cancerous prostates. This approach not only provides additional valuable information not accessible by standard clinical histopathological assignment but can also be used for improved diagnostics of PC. In addition, the outcome of our study provides clear evidence for the TINT concept that tumor indicating normal tissues are also affected at the metabolomic level. Moreover, the degree of metabolomic alteration reflects even the aggressiveness of neighbouring tumor and the distance from it. Therefore, the HR MAS NMR analysis approach of prostate biopsies may have important clinical potential for identifying cancer and its aggressive state in benign biopsies from cancerous prostates. Understanding the dynamics and mechanisms driving the cross-talk between tumor and non-cancerous “benign” tissue in the affected prostate aid in understanding prostate carcinogenesis, progression, recurrence, and resistance to treatment. This can provide new opportunities for the diagnosis and prognostication of PC patients.

## Data Availability

The raw data supporting the conclusions of this article will be made available by the authors, without undue reservation.
