# Supplementary material for: Metabolic readouts of tumor instructed normal tissues (TINT) identify aggressive prostate cancer subgroups for tailored therapy
Source: Front Mol Biosci. 2025 Apr 7;12:1426949. doi: 10.3389/fmolb.2025.1426949 (PMC12009692; doi:10.3389/fmolb.2025.1426949)
Supplement: Supplementary file 1 [file Table1.docx]

Table S1. Metabolites identified by ^1^H HR MAS NMR in prostate tissue samples.

| **Nr** | **Metabolite** | **Chemical shift (ppm)** |
| --- | --- | --- |
| 1 | Lipids –CH_3_ | 0.87 |
| 2 | Isoleucine | 0.93, **1.01** |
| 3 | Leucine | 0.93, **0.95,** 1.68, 1.79 |
| 4 | Valine | 0.95, 0.98, **1.04** |
| 5 | Lipids –CH_2_ | 1.26 |
| 6 | Lactate | 1.34, **4.12** |
| 7 | Alanine | **1.47**, 3.76 |
| 8 | Lipids –CH_2_–CH_2_–C=O | 1.59 |
| 9 | Arginine | 1.69. 1.79, **1.87,** 1.92, 3.76 |
| 10 | Lysine | 1.69, **1.79**, 1.87, 1.92 |
| 11 | Polyamines | 1.79, **3.09** |
| 12 | Acetate | 1.92 |
| 13 | Lipids –CH_2_–CH= | 2.07 |
| 14 | Glutamate | 2.07, **2.34**, 3.76 |
| 15 | Glutamine | 2.07, **2.46**, 3.76 |
| 16 | Methionine | 2.07, **2.64,** 2.66 |
| 17 | Lipids –CH_2_–CH_2_–C=O | 2.25 |
| 18 | Pyruvate | 2.37 |
| 19 | Succinate | 2.38 |
| 20 | 2-Oxoglutarate | **2.46**, 2.99 |
| 21 | Citrate | **2.54**, 2.71 |
| 22 | Aspartate | 2.66, 2.71, **2.81**, 3.89 |
| 23 | Malate | 2.66 |
| 24 | Lipids =CH–CH_2_–CH= | 2.81 |
| 25 | Asparagine | **2.87**, 2.95 |
| 26 | Dimethylglycine | 2.91 |
| 27 | Glutathione | 2.95, 2.99, **4.58** |
| 28 | Creatine | **3.02,** 3.93 |
| 29 | Ethanolamine | 3.14, **3.81** |
| 30 | Choline | **3.19**, 3.53 |
| 31 | Phosphocholine | **3.22**, 3.60, 4.18 |
| 32 | Glycero-3-phosphocholine | **3.22**, 3.89, 4.32 |
| 33 | Taurine | 3.26, 3.29, **3.42** |
| 34 | Myo-inositol | 3.29, 3.53, 3.56, 3.60, **4.06** |
| 35 | Scyllo-inositol | 3.34 |
| 36 | Glycerol | 3.53, 3.56, **3.57,** 3.65, 3.76 |
| 37 | Glycine | 3.56 |
| 38 | Threonine | 1.34, 3.57, **4.26** |
| 39 | Glucose | 3.73, **4.65** |
| 40 | Ascorbate | 3.76, **4.52** |
| 41 | Serine | 3.85, **3.98** |
| 42 | Threonine | 4.26 |
| 43 | Inosine | **6.09**, 8.23, 8.35 |
| 44 | Fumarate | 6.52 |
| 45 | Tyrosine | **6.88**, 7.17 |
| 46 | Tryptophan | 7.20, 7.31, 7.73 |
| 47 | Phenylalanine | 7.31, 7.36, **7.41** |
| 48 | Hypoxanthine | 8.17 |

*In bold are marked ppm values of the main peaks used for identification of selected metabolites.
