# Supplementary figures and images for: Metabolic readouts of tumor instructed normal tissues (TINT) identify aggressive prostate cancer subgroups for tailored therapy
Source: Front Mol Biosci. 2025 Apr 7;12:1426949. doi: 10.3389/fmolb.2025.1426949 (PMC12009692; doi:10.3389/fmolb.2025.1426949)

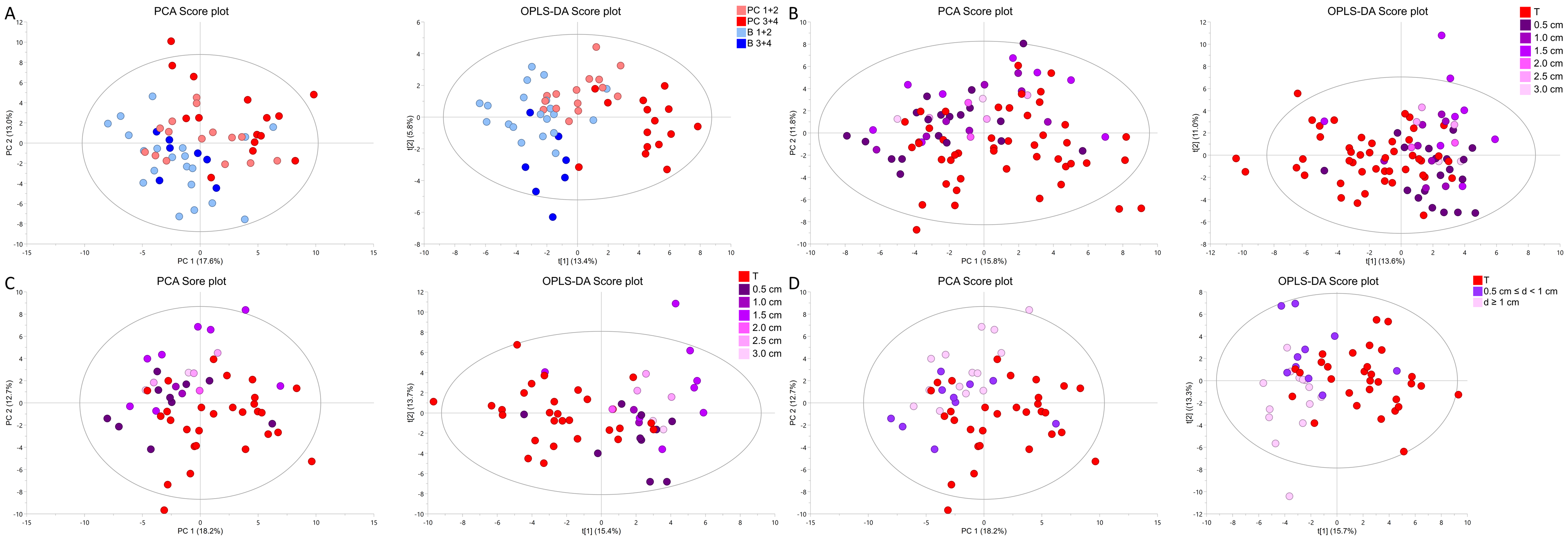

Supplement: Supplementary file 2 [file Image3.tif]

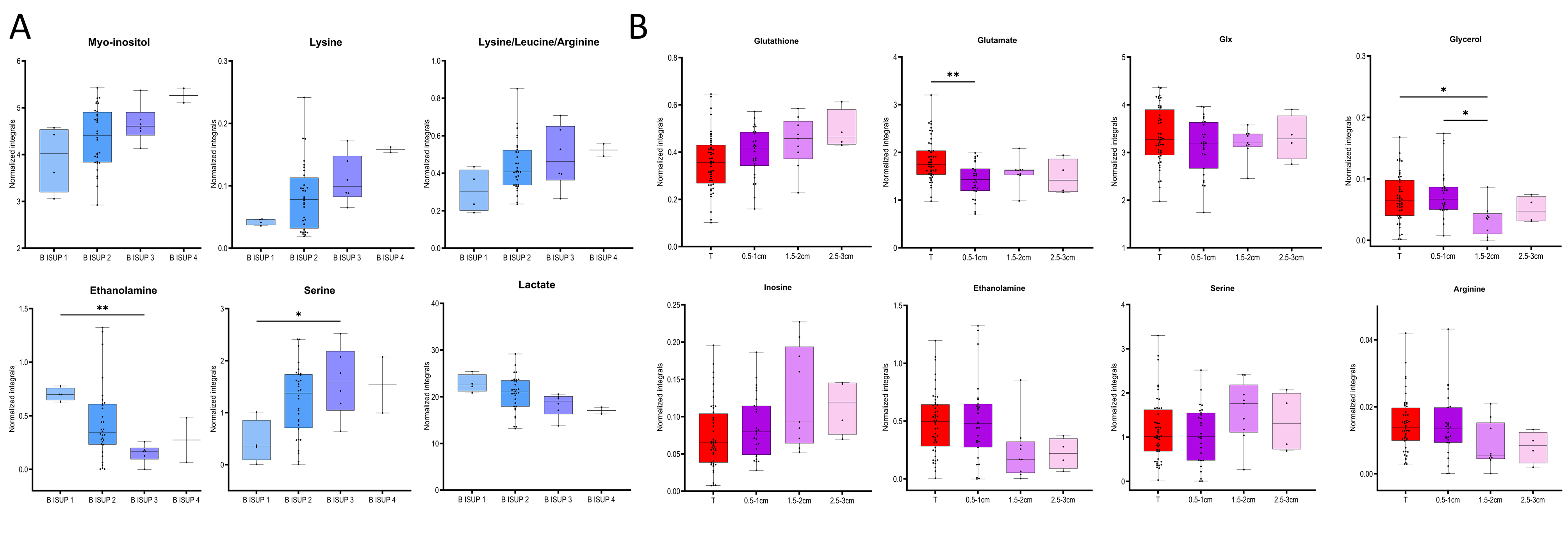

Supplement: Supplementary file 3 [file Image4.tif]

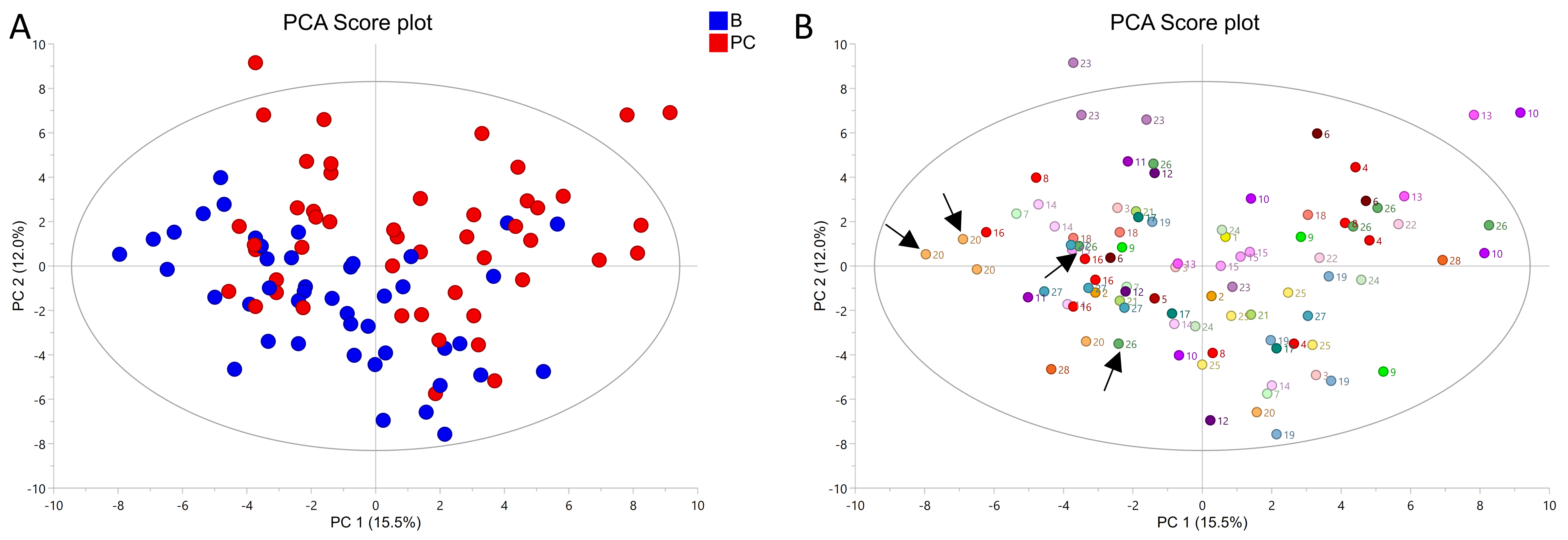

Supplement: Supplementary file 4 [file Image2.tif]

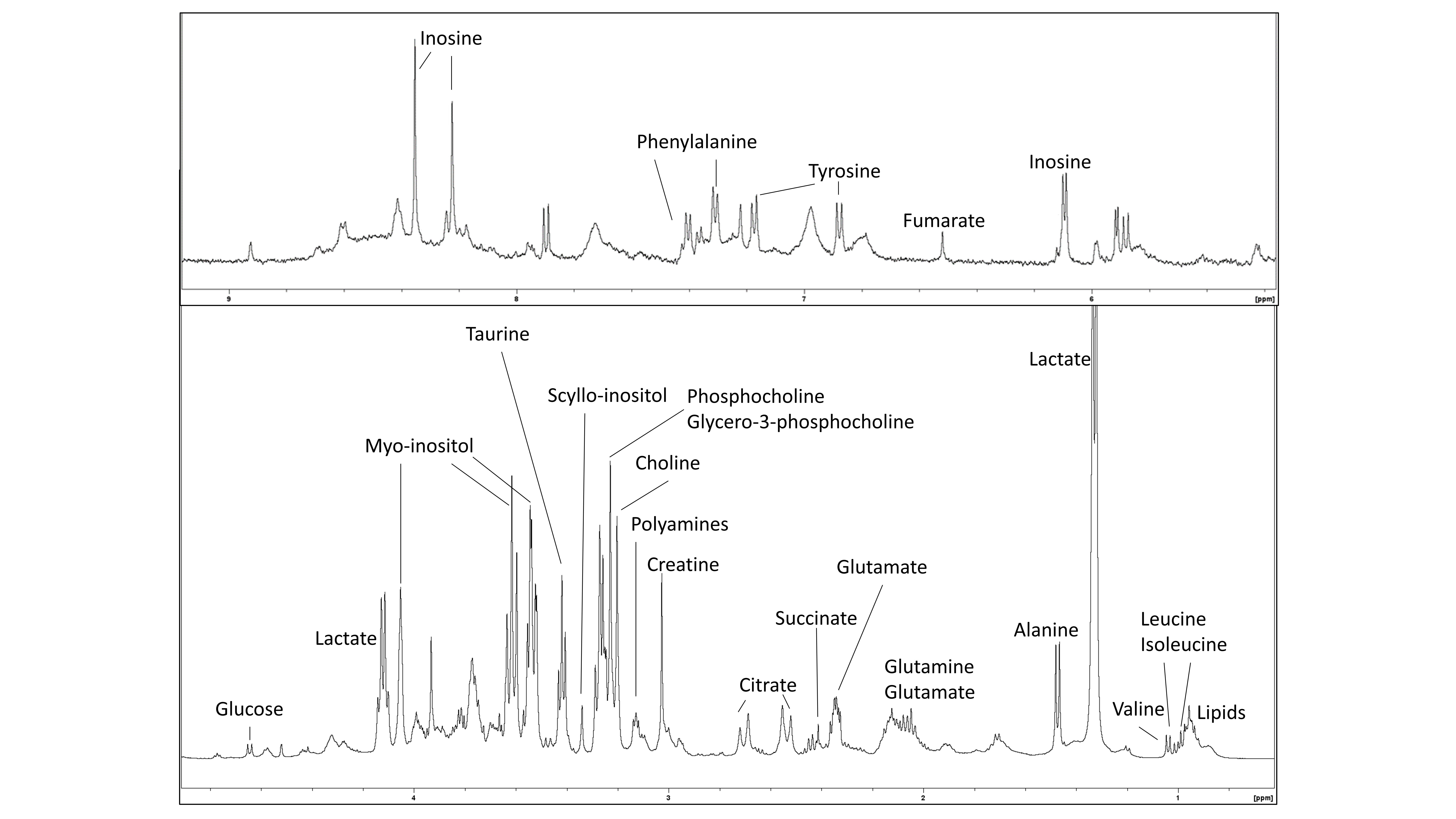

Supplement: Supplementary file 6 [file Image1.tif]
