# Supplementary material for: Metabolic readouts of tumor instructed normal tissues (TINT) identify aggressive prostate cancer subgroups for tailored therapy
Source: Front Mol Biosci. 2025 Apr 7;12:1426949. doi: 10.3389/fmolb.2025.1426949 (PMC12009692; doi:10.3389/fmolb.2025.1426949)
Supplement: Supplementary file 5 [file Table7.docx]

**Table S7. Comparison of benign samples based on the distance from the closest tumor from PC patients with unifocal tumors only (n = 24) for all integrated variables.**

| **Nr** | **Chemical shift (ppm)** | **Correlation with distance from the tumor** | | | **0.5 cm ≤ d < 1 cm**  ***vs* d ≥ 1 cm** | | **Post-hoc analysis 0.5 cm ≤ d < 1 cm**  ***vs* d ≥ 1 cm** | |
| --- | --- | --- | --- | --- | --- | --- | --- | --- |
|  |  | **coefficient** | ***p*-value** | **q value*** | ***p*-value** | **q value*** | ***p*-value** | **q value*** |
| 1 | 0.87 | 0.1244 | 0.5625 | 0.9802 | 0.3340 | 0.7690 | 0.8185 | 0.9999 |
| 2 | 0.93 | -0.0284 | 0.8953 | 0.9802 | 0.4590 | 0.7690 | 0.7120 | 0.9999 |
| 3 | 0.96 | -0.1109 | 0.6058 | 0.9802 | 0.9766 | 0.9874 | 0.9999 | 0.9999 |
| 4 | 0.99 | -0.0837 | 0.6973 | 0.9802 | 0.5854 | 0.7690 | 0.8455 | 0.9999 |
| 5 | 1.01 | -0.1005 | 0.6404 | 0.9802 | 0.3641 | 0.7690 | 0.9999 | 0.9999 |
| 6 | 1.04 | 0.0164 | 0.9393 | 0.9820 | 0.5007 | 0.7690 | 0.9999 | 0.9999 |
| 7 | 1.26 | 0.0587 | 0.7854 | 0.9802 | 0.1207 | 0.5049 | 0.4127 | 0.9999 |
| 8 | 1.34 | -0.2352 | 0.2685 | 0.9802 | 0.1360 | 0.5439 | 0.3347 | 0.9999 |
| 9 | 1.41 | 0.1151 | 0.5924 | 0.9802 | 0.9300 | 0.9835 | 0.9999 | 0.9999 |
| 10 | 1.45 | -0.2485 | 0.2417 | 0.9802 | 0.0525 | 0.4373 | 0.2484 | 0.9999 |
| 11 | 1.47 | 0.1642 | 0.4432 | 0.9802 | 0.3814 | 0.7690 | 0.7309 | 0.9999 |
| 12 | 1.59 | 0.0630 | 0.7701 | 0.9802 | **0.0077** | 0.2555 | **0.0025** | 0.2300 |
| 13 | 1.69 | -0.0302 | 0.8888 | 0.9802 | 0.8004 | 0.8981 | 0.9705 | 0.9999 |
| 14 | 1.79 | -0.1275 | 0.5529 | 0.9802 | 0.5387 | 0.7690 | 0.9999 | 0.9999 |
| 15 | 1.88 | -0.3060 | 0.1459 | 0.9672 | 0.1688 | 0.5942 | 0.5893 | 0.9999 |
| 16 | 1.92 | 0.0776 | 0.7186 | 0.9802 | 0.1073 | 0.4703 | 0.1430 | 0.9999 |
| 17 | 2.08 | 0.0375 | 0.8618 | 0.9802 | 0.3055 | 0.7596 | 0.9999 | 0.9999 |
| 18 | 2.25 | 0.0625 | 0.7718 | 0.9802 | 0.4292 | 0.7690 | 0.9999 | 0.9999 |
| 19 | 2.30 | -0.1806 | 0.3983 | 0.9802 | 0.2350 | 0.6551 | 0.5171 | 0.9999 |
| 20 | 2.34 | 0.0892 | 0.6784 | 0.9802 | 0.3959 | 0.7690 | 0.9999 | 0.9999 |
| 21 | 2.37 | -0.3706 | 0.0746 | 0.9672 | **0.0171** | 0.2555 | **0.0456** | 0.8295 |
| 22 | 2.38 | -0.2231 | 0.2946 | 0.9802 | **0.0417** | 0.4264 | 0.1057 | 0.9999 |
| 23 | 2.42 | 0.0470 | 0.8275 | 0.9802 | 0.3762 | 0.7690 | 0.6754 | 0.9999 |
| 24 | 2.46 | 0.0430 | 0.8419 | 0.9802 | 0.6264 | 0.7690 | 0.8697 | 0.9999 |
| 25 | 2.55 | -0.0605 | 0.7789 | 0.9802 | 0.9766 | 0.9874 | 0.9999 | 0.9999 |
| 26 | 2.64 | 0.0544 | 0.8008 | 0.9802 | 0.6187 | 0.7690 | 0.9999 | 0.9999 |
| 27 | 2.66 | -0.1354 | 0.5282 | 0.9802 | 0.6023 | 0.7690 | 0.8775 | 0.9999 |
| 28 | 2.71 | -0.1489 | 0.4873 | 0.9802 | 0.5780 | 0.7690 | 0.9999 | 0.9999 |
| 29 | 2.76 | 0.0960 | 0.6555 | 0.9802 | **0.0482** | 0.4373 | 0.1249 | 0.9999 |
| 30 | 2.81 | 0.0868 | 0.6869 | 0.9802 | 0.3959 | 0.7690 | 0.9999 | 0.9999 |
| 31 | 2.87 | -0.0303 | 0.8881 | 0.9802 | 0.3340 | 0.7690 | 0.9250 | 0.9999 |
| 32 | 2.91 | 0.1457 | 0.4968 | 0.9802 | 0.6187 | 0.7690 | 0.9999 | 0.9999 |
| 33 | 2.95 | 0.3065 | 0.1452 | 0.9672 | 0.1031 | 0.4703 | 0.2104 | 0.9999 |
| 34 | 2.99 | 0.1101 | 0.6087 | 0.9802 | 0.5618 | 0.7690 | 0.7801 | 0.9999 |
| 35 | 3.02 | -0.0050 | 0.9814 | 0.9848 | 0.6537 | 0.7913 | 0.8676 | 0.9999 |
| 36 | 3.05 | -0.1411 | 0.5107 | 0.9802 | 0.1762 | 0.5942 | 0.4222 | 0.9999 |
| 37 | 3.09 | -0.1690 | 0.4298 | 0.9802 | 0.5780 | 0.7690 | 0.9999 | 0.9999 |
| 38 | 3.14 | -0.4143 | **0.0441** | 0.9672 | 0.1073 | 0.4703 | 0.2751 | 0.9999 |
| 39 | 3.19 | -0.0712 | 0.7411 | 0.9802 | 0.7922 | 0.8981 | 0.9999 | 0.9999 |
| 40 | 3.22 | -0.0666 | 0.7571 | 0.9802 | 0.8836 | 0.9564 | 0.9999 | 0.9999 |
| 41 | 3.26 | 0.0901 | 0.6756 | 0.9802 | 0.6269 | 0.7690 | 0.8498 | 0.9999 |
| 42 | 3.29 | -0.2240 | 0.2928 | 0.9802 | 0.2642 | 0.6946 | 0.3868 | 0.9999 |
| 43 | 3.34 | -0.1731 | 0.4186 | 0.9802 | 0.3055 | 0.7596 | 0.9807 | 0.9999 |
| 44 | 3.42 | 0.2254 | 0.2895 | 0.9802 | 0.1860 | 0.5942 | 0.3468 | 0.9999 |
| 45 | 3.48 | 0.0680 | 0.7524 | 0.9802 | 0.7474 | 0.8704 | 0.9999 | 0.9999 |
| 46 | 3.53 | 0.0183 | 0.9323 | 0.9820 | 0.5552 | 0.7690 | 0.8781 | 0.9999 |
| 47 | 3.56 | -0.2537 | 0.2316 | 0.9802 | 0.1873 | 0.5942 | 0.4229 | 0.9999 |
| 48 | 3.57 | -0.3913 | 0.0587 | 0.9672 | **0.0075** | 0.2555 | **0.0189** | 0.4416 |
| 49 | 3.60 | -0.0441 | 0.8380 | 0.9802 | 0.4330 | 0.7690 | 0.7517 | 0.9999 |
| 50 | 3.69 | -0.2321 | 0.2751 | 0.9802 | 0.4292 | 0.7690 | 0.9999 | 0.9999 |
| 51 | 3.71 | -0.0041 | 0.9848 | 0.9848 | 0.5387 | 0.7690 | 0.9999 | 0.9999 |
| 52 | 3.73 | -0.0254 | 0.9062 | 0.9802 | 0.7922 | 0.8981 | 0.9999 | 0.9999 |
| 53 | 3.76 | 0.3086 | 0.1423 | 0.9672 | 0.0712 | 0.4546 | 0.3282 | 0.9999 |
| 54 | 3.81 | -0.4014 | 0.0519 | 0.9672 | 0.0841 | 0.4703 | 0.3282 | 0.9999 |
| 55 | 3.85 | -0.0241 | 0.9111 | 0.9802 | 0.9300 | 0.9835 | 0.9999 | 0.9999 |
| 56 | 3.89 | -0.0643 | 0.7654 | 0.9802 | 0.1688 | 0.5942 | 0.6290 | 0.9999 |
| 57 | 3.93 | 0.0299 | 0.8897 | 0.9802 | 0.5007 | 0.7690 | 0.9999 | 0.9999 |
| 58 | 3.98 | 0.2555 | 0.2283 | 0.9802 | 0.0741 | 0.4546 | 0.1832 | 0.9999 |
| 59 | 4.06 | 0.0644 | 0.7650 | 0.9802 | 0.2530 | 0.6846 | 0.5881 | 0.9999 |
| 60 | 4.12 | -0.1564 | 0.4656 | 0.9802 | 0.1029 | 0.4703 | 0.3038 | 0.9999 |
| 61 | 4.18 | -0.2096 | 0.3256 | 0.9802 | 0.6184 | 0.7690 | 0.9999 | 0.9999 |
| 62 | 4.21 | 0.3117 | 0.1382 | 0.9672 | 0.5981 | 0.7690 | 0.9999 | 0.9999 |
| 63 | 4.26 | 0.0048 | 0.9824 | 0.9848 | 0.8792 | 0.9564 | 0.9923 | 0.9999 |
| 64 | 4.32 | -0.2505 | 0.2378 | 0.9802 | 0.2300 | 0.6551 | 0.9203 | 0.9999 |
| 65 | 4.41 | 0.1378 | 0.5207 | 0.9802 | 0.6187 | 0.7690 | 0.9999 | 0.9999 |
| 66 | 4.44 | 0.1086 | 0.6134 | 0.9802 | 0.1688 | 0.5942 | 0.5825 | 0.9999 |
| 67 | 4.52 | 0.1213 | 0.5722 | 0.9802 | 0.5387 | 0.7690 | 0.9999 | 0.9999 |
| 68 | 4.58 | 0.4458 | **0.0290** | 0.9672 | **0.0222** | 0.2555 | 0.0541 | 0.8295 |
| 69 | 4.65 | 0.0739 | 0.7316 | 0.9802 | 0.3641 | 0.7690 | 0.9999 | 0.9999 |
| 70 | 5.88 | 0.2374 | 0.2640 | 0.9802 | 0.3959 | 0.7690 | 0.7487 | 0.9999 |
| 71 | 5.92 | 0.3035 | 0.1494 | 0.9672 | **0.0207** | 0.2555 | 0.0809 | 0.9999 |
| 72 | 5.97 | 0.0936 | 0.6636 | 0.9802 | 0.9766 | 0.9874 | 0.9999 | 0.9999 |
| 73 | 6.09 | 0.3306 | 0.1146 | 0.9672 | **0.0114** | 0.2555 | **0.0125** | 0.4416 |
| 74 | 6.52 | 0.2393 | 0.2601 | 0.9802 | 0.2081 | 0.6381 | 0.7678 | 0.9999 |
| 75 | 6.61 | -0.0402 | 0.8520 | 0.9802 | 0.5780 | 0.7690 | 0.9999 | 0.9999 |
| 76 | 6.79 | -0.1708 | 0.4249 | 0.9802 | 0.5780 | 0.7690 | 0.9999 | 0.9999 |
| 77 | 6.88 | 0.0297 | 0.8905 | 0.9802 | 0.4292 | 0.7690 | 0.9999 | 0.9999 |
| 78 | 6.99 | 0.0412 | 0.8483 | 0.9802 | 0.8420 | 0.9332 | 0.9709 | 0.9999 |
| 79 | 7.17 | 0.0107 | 0.9606 | 0.9848 | 0.5387 | 0.7690 | 0.9999 | 0.9999 |
| 80 | 7.20 | 0.0227 | 0.9163 | 0.9802 | 0.9766 | 0.9874 | 0.9999 | 0.9999 |
| 81 | 7.31 | -0.2066 | 0.3329 | 0.9802 | 0.6187 | 0.7690 | 0.9999 | 0.9999 |
| 82 | 7.36 | 0.1647 | 0.4420 | 0.9802 | 0.4292 | 0.7690 | 0.9999 | 0.9999 |
| 83 | 7.41 | -0.0631 | 0.7697 | 0.9802 | 1.0000 | 1.0000 | 0.9999 | 0.9999 |
| 84 | 7.73 | 0.0446 | 0.8362 | 0.9802 | 0.5894 | 0.7690 | 0.8492 | 0.9999 |
| 85 | 7.90 | 0.2622 | 0.2158 | 0.9802 | 0.0570 | 0.4373 | 0.2205 | 0.9999 |
| 86 | 7.96 | 0.0306 | 0.8872 | 0.9802 | 0.7474 | 0.8704 | 0.9999 | 0.9999 |
| 87 | 8.17 | -0.4007 | 0.0523 | 0.9672 | 0.0741 | 0.4546 | 0.3694 | 0.9999 |
| 88 | 8.23 | 0.3344 | 0.1103 | 0.9672 | **0.0151** | 0.2555 | 0.1018 | 0.9999 |
| 89 | 8.35 | 0.2977 | 0.1577 | 0.9672 | **0.0153** | 0.2555 | **0.0192** | 0.4416 |
| 90 | 8.41 | 0.0894 | 0.6777 | 0.9802 | 0.7120 | 0.8507 | 0.9314 | 0.9999 |
| 91 | 8.60 | -0.1309 | 0.5421 | 0.9802 | 0.2300 | 0.6551 | 0.4716 | 0.9999 |
| 92 | 8.93 | 0.3380 | 0.1062 | 0.9672 | 0.1073 | 0.4703 | 0.3138 | 0.9999 |

q-value is based on Benjamini-Hochberg correction.
