# Supplementary material for: Metabolic readouts of tumor instructed normal tissues (TINT) identify aggressive prostate cancer subgroups for tailored therapy
Source: Front Mol Biosci. 2025 Apr 7;12:1426949. doi: 10.3389/fmolb.2025.1426949 (PMC12009692; doi:10.3389/fmolb.2025.1426949)
Supplement: Supplementary file 7 [file Table2.docx]

**Table S2. Comparison of benign samples accompanying tumors with ISUP 1+2 and ISUP 3+4 from PC patients with unifocal and selected multifocal tumors (n = 44)** **for all integrated variables.**

| **Nr** | **Chemical shift (ppm)** | **Correlation with ISUP values** | | | **B ISUP 1+2 *vs* B ISUP 3+4** | | **Post-hoc analysis B ISUP 1+2 *vs***  **B ISUP 3+4** | |
| --- | --- | --- | --- | --- | --- | --- | --- | --- |
|  |  | **coefficient** | ***p*-value** | **q value*** | ***p*-value** | **q value*** | ***p*-value** | **q value*** |
| 1 | 0.87 | 0.06 | 0.6781 | 0.9520 | 0.8432 | 0.9972 | 0.9999 | 0.9999 |
| 2 | 0.93 | 0.05 | 0.7347 | 0.9520 | 0.7161 | 0.9972 | 0.9794 | 0.9999 |
| 3 | 0.96 | -0.05 | 0.7252 | 0.9520 | 0.5529 | 0.9765 | 0.9999 | 0.9999 |
| 4 | 0.99 | -0.08 | 0.5961 | 0.9495 | 0.3288 | 0.8176 | 0.748 | 0.9999 |
| 5 | 1.01 | 0.06 | 0.7221 | 0.9520 | 0.9394 | 0.9987 | 0.9999 | 0.9999 |
| 6 | 1.04 | -0.01 | 0.9275 | 0.9753 | 0.7959 | 0.9972 | 0.9999 | 0.9999 |
| 7 | 1.26 | 0.02 | 0.9009 | 0.9753 | 0.4378 | 0.8950 | 0.9999 | 0.9999 |
| 8 | 1.34 | -0.36 | **0.0165** | 0.1519 | **0.0373** | 0.3665 | 0.1263 | 0.9999 |
| 9 | 1.41 | 0.42 | **0.0041** | 0.1125 | **0.0398** | 0.3665 | 0.117 | 0.9999 |
| 10 | 1.45 | -0.21 | 0.1741 | 0.6408 | 0.1959 | 0.6676 | 0.9999 | 0.9999 |
| 11 | 1.47 | -0.08 | 0.6148 | 0.9495 | 0.8195 | 0.9972 | 0.9999 | 0.9999 |
| 12 | 1.59 | 0.03 | 0.8456 | 0.9753 | 0.1571 | 0.6676 | 0.9999 | 0.9999 |
| 13 | 1.69 | 0.31 | **0.0413** | 0.3168 | 0.1662 | 0.6676 | 0.9999 | 0.9999 |
| 14 | 1.79 | -0.16 | 0.2960 | 0.7781 | 0.9636 | 0.9987 | 0.9999 | 0.9999 |
| 15 | 1.88 | -0.03 | 0.8630 | 0.9753 | 0.2938 | 0.8176 | 0.9999 | 0.9999 |
| 16 | 1.92 | -0.07 | 0.6578 | 0.9520 | 1.0000 | 1.0000 | 0.9999 | 0.9999 |
| 17 | 2.08 | 0.24 | 0.1165 | 0.5276 | **0.0319** | 0.3665 | 0.331 | 0.9999 |
| 18 | 2.25 | 0.01 | 0.9490 | 0.9753 | 0.7725 | 0.9972 | 0.9999 | 0.9999 |
| 19 | 2.30 | 0.17 | 0.2595 | 0.7614 | 0.6354 | 0.9972 | 0.9999 | 0.9999 |
| 20 | 2.34 | 0.19 | 0.2102 | 0.6907 | 0.5652 | 0.9765 | 0.9446 | 0.9999 |
| 21 | 2.37 | -0.17 | 0.2783 | 0.7758 | 0.2384 | 0.7832 | 0.6312 | 0.9999 |
| 22 | 2.38 | -0.12 | 0.4509 | 0.9018 | 0.2883 | 0.8176 | 0.7146 | 0.9999 |
| 23 | 2.42 | -0.21 | 0.1728 | 0.6408 | 0.5944 | 0.9765 | 0.9999 | 0.9999 |
| 24 | 2.46 | 0.19 | 0.2084 | 0.6907 | 0.7697 | 0.9972 | 0.9888 | 0.9999 |
| 25 | 2.55 | 0.15 | 0.3438 | 0.8498 | 0.4200 | 0.8950 | 0.9999 | 0.9999 |
| 26 | 2.64 | -0.02 | 0.9224 | 0.9753 | 0.9879 | 0.9987 | 0.9999 | 0.9999 |
| 27 | 2.66 | -0.02 | 0.9139 | 0.9753 | 0.8671 | 0.9972 | 0.9999 | 0.9999 |
| 28 | 2.71 | 0.06 | 0.6794 | 0.9520 | 0.5130 | 0.9631 | 0.9999 | 0.9999 |
| 29 | 2.76 | -0.06 | 0.6984 | 0.9520 | 0.4493 | 0.8986 | 0.9999 | 0.9999 |
| 30 | 2.81 | 0.14 | 0.3787 | 0.8498 | 0.7493 | 0.9972 | 0.9999 | 0.9999 |
| 31 | 2.87 | 0.11 | 0.4758 | 0.9119 | 0.9636 | 0.9987 | 0.9999 | 0.9999 |
| 32 | 2.91 | 0.28 | 0.0615 | 0.3775 | 0.0613 | 0.4699 | 0.5647 | 0.9999 |
| 33 | 2.95 | 0.02 | 0.9015 | 0.9753 | 0.3231 | 0.8176 | 0.7868 | 0.9999 |
| 34 | 2.99 | 0.43 | **0.0038** | 0.1125 | **0.0234** | 0.3665 | 0.0627 | 0.9999 |
| 35 | 3.02 | -0.14 | 0.3628 | 0.8498 | 0.1171 | 0.6392 | 0.687 | 0.9999 |
| 36 | 3.05 | 0.08 | 0.6172 | 0.9495 | 0.6817 | 0.9972 | 0.9999 | 0.9999 |
| 37 | 3.09 | -0.05 | 0.7628 | 0.9723 | 0.5529 | 0.9765 | 0.9999 | 0.9999 |
| 38 | 3.14 | -0.25 | 0.1008 | 0.4879 | 0.4027 | 0.8821 | 0.9999 | 0.9999 |
| 39 | 3.19 | 0.22 | 0.1460 | 0.6020 | 0.2938 | 0.8176 | 0.9999 | 0.9999 |
| 40 | 3.22 | 0.08 | 0.6204 | 0.9495 | 0.5328 | 0.9765 | 0.9999 | 0.9999 |
| 41 | 3.26 | 0.07 | 0.6295 | 0.9495 | 0.6942 | 0.9972 | 0.9748 | 0.9999 |
| 42 | 3.29 | 0.00 | 0.9917 | 0.9917 | 0.6753 | 0.9972 | 0.9684 | 0.9999 |
| 43 | 3.34 | -0.09 | 0.5552 | 0.9495 | 0.4746 | 0.9289 | 0.9999 | 0.9999 |
| 44 | 3.42 | -0.01 | 0.9270 | 0.9753 | 0.3892 | 0.8821 | 0.7827 | 0.9999 |
| 45 | 3.48 | 0.04 | 0.7773 | 0.9723 | 0.5735 | 0.9765 | 0.9999 | 0.9999 |
| 46 | 3.53 | 0.25 | 0.0953 | 0.4870 | 0.1855 | 0.6676 | 0.6328 | 0.9999 |
| 47 | 3.56 | -0.09 | 0.5656 | 0.9495 | 0.7036 | 0.9972 | 0.9999 | 0.9999 |
| 48 | 3.57 | -0.28 | 0.0616 | 0.3775 | 0.1757 | 0.6676 | 0.8062 | 0.9999 |
| 49 | 3.60 | 0.29 | 0.0528 | 0.3737 | 0.3147 | 0.8176 | 0.7535 | 0.9999 |
| 50 | 3.69 | 0.00 | 0.9898 | 0.9917 | 0.8432 | 0.9972 | 0.9999 | 0.9999 |
| 51 | 3.71 | 0.14 | 0.3747 | 0.8498 | 0.7493 | 0.9972 | 0.9999 | 0.9999 |
| 52 | 3.73 | -0.27 | 0.0797 | 0.4311 | 0.1320 | 0.6392 | 0.7477 | 0.9999 |
| 53 | 3.76 | 0.20 | 0.1852 | 0.6554 | 0.3791 | 0.8821 | 0.8274 | 0.9999 |
| 54 | 3.81 | -0.37 | **0.0143** | 0.1519 | **0.0131** | 0.3665 | 0.0642 | 0.9999 |
| 55 | 3.85 | -0.12 | 0.4393 | 0.9018 | 0.1320 | 0.6392 | 0.9017 | 0.9999 |
| 56 | 3.89 | -0.16 | 0.2952 | 0.7781 | 0.4027 | 0.8821 | 0.9999 | 0.9999 |
| 57 | 3.93 | -0.13 | 0.3880 | 0.8498 | 0.0802 | 0.5674 | 0.4486 | 0.9999 |
| 58 | 3.98 | 0.35 | **0.0197** | 0.1644 | 0.1096 | 0.6392 | 0.3779 | 0.9999 |
| 59 | 4.06 | 0.39 | **0.0083** | 0.1519 | **0.0472** | 0.3950 | 0.2168 | 0.9999 |
| 60 | 4.12 | -0.36 | **0.0159** | 0.1519 | **0.0355** | 0.3665 | 0.1777 | 0.9999 |
| 61 | 4.18 | 0.04 | 0.7821 | 0.9723 | 0.1956 | 0.6676 | 0.9999 | 0.9999 |
| 62 | 4.21 | -0.06 | 0.7219 | 0.9520 | 0.0971 | 0.6382 | 0.6684 | 0.9999 |
| 63 | 4.26 | -0.03 | 0.8642 | 0.9753 | 0.2800 | 0.8176 | 0.9999 | 0.9999 |
| 64 | 4.32 | -0.17 | 0.2646 | 0.7614 | 0.3080 | 0.8176 | 0.9999 | 0.9999 |
| 65 | 4.41 | -0.01 | 0.9308 | 0.9753 | 0.6812 | 0.9972 | 0.9999 | 0.9999 |
| 66 | 4.44 | 0.37 | **0.0122** | 0.1519 | 0.7264 | 0.9972 | 0.9999 | 0.9999 |
| 67 | 4.52 | 0.17 | 0.2648 | 0.7614 | 0.5130 | 0.9631 | 0.9999 | 0.9999 |
| 68 | 4.58 | 0.12 | 0.4443 | 0.9018 | 0.7193 | 0.9972 | 0.9849 | 0.9999 |
| 69 | 4.65 | -0.19 | 0.2223 | 0.7051 | 0.4378 | 0.8950 | 0.9999 | 0.9999 |
| 70 | 5.88 | -0.10 | 0.5371 | 0.9495 | 0.1757 | 0.6676 | 0.9999 | 0.9999 |
| 71 | 5.92 | 0.08 | 0.6128 | 0.9495 | 0.9394 | 0.9987 | 0.9999 | 0.9999 |
| 72 | 5.97 | 0.01 | 0.9347 | 0.9753 | 0.8432 | 0.9972 | 0.9999 | 0.9999 |
| 73 | 6.09 | 0.15 | 0.3376 | 0.8498 | 0.9879 | 0.9987 | 0.9999 | 0.9999 |
| 74 | 6.52 | 0.11 | 0.4645 | 0.9093 | 0.8671 | 0.9972 | 0.9999 | 0.9999 |
| 75 | 6.61 | 0.57 | **0.0001** | 0.0049 | **0.0030** | 0.2119 | **0.019** | 0.874 |
| 76 | 6.79 | -0.27 | 0.0744 | 0.4276 | **0.0274** | 0.3665 | 0.1333 | 0.9999 |
| 77 | 6.88 | 0.06 | 0.6792 | 0.9520 | 0.9879 | 0.9987 | 0.9999 | 0.9999 |
| 78 | 6.99 | 0.37 | **0.0136** | 0.1519 | **0.0185** | 0.3665 | 0.1049 | 0.9999 |
| 79 | 7.17 | 0.08 | 0.5987 | 0.9495 | 0.3694 | 0.8821 | 0.9999 | 0.9999 |
| 80 | 7.20 | 0.05 | 0.7300 | 0.9520 | 0.1244 | 0.6392 | 0.9176 | 0.9999 |
| 81 | 7.31 | -0.22 | 0.1505 | 0.6020 | 0.1662 | 0.6676 | 0.9999 | 0.9999 |
| 82 | 7.36 | 0.24 | 0.1204 | 0.5276 | 0.5944 | 0.9765 | 0.9999 | 0.9999 |
| 83 | 7.41 | 0.01 | 0.9541 | 0.9753 | 0.9394 | 0.9987 | 0.9999 | 0.9999 |
| 84 | 7.73 | 0.03 | 0.8636 | 0.9753 | 0.7179 | 0.9972 | 0.9822 | 0.9999 |
| 85 | 7.90 | 0.13 | 0.3976 | 0.8507 | 0.9636 | 0.9987 | 0.9999 | 0.9999 |
| 86 | 7.96 | -0.04 | 0.8021 | 0.9753 | 0.8432 | 0.9972 | 0.9999 | 0.9999 |
| 87 | 8.17 | 0.08 | 0.5963 | 0.9495 | 0.2938 | 0.8176 | 0.9999 | 0.9999 |
| 88 | 8.23 | 0.10 | 0.5201 | 0.9495 | 0.9152 | 0.9987 | 0.9999 | 0.9999 |
| 89 | 8.35 | 0.14 | 0.3695 | 0.8498 | 0.9394 | 0.9987 | 0.999 | 0.9999 |
| 90 | 8.41 | 0.10 | 0.5175 | 0.9495 | 0.8600 | 0.9972 | 0.9985 | 0.9999 |
| 91 | 8.60 | -0.42 | **0.0049** | 0.1125 | **0.0046** | 0.2119 | **0.0113** | 0.874 |
| 92 | 8.93 | -0.04 | 0.8085 | 0.9753 | 0.6812 | 0.9972 | 0.9999 | 0.9999 |

q-value is based on Benjamini-Hochberg correction.
