# Supplementary material for: Metabolic readouts of tumor instructed normal tissues (TINT) identify aggressive prostate cancer subgroups for tailored therapy
Source: Front Mol Biosci. 2025 Apr 7;12:1426949. doi: 10.3389/fmolb.2025.1426949 (PMC12009692; doi:10.3389/fmolb.2025.1426949)
Supplement: Supplementary file 8 [file Table5.docx]

**Table S5. Comparison of tumor samples with ISUP 1+2 and ISUP 3+4 from PC patients with unifocal tumors only (n = 30) for all integrated variables.**

| **Nr** | **Chemical shift (ppm)** | **Correlation with ISUP values** | | | **PC ISUP 1+2 *vs* PC ISUP 3+4** | | **Post-hoc analysis PC ISUP 1+2 *vs***  **PC ISUP 3+4** | |
| --- | --- | --- | --- | --- | --- | --- | --- | --- |
|  |  | **coefficient** | ***p*-value** | **q value*** | ***p*-value** | **q value*** | ***p*-value** | **q value*** |
| 1 | 0.87 | -0.1119 | 0.5561 | 0.7105 | 0.2455 | 0.5133 | 0.9999 | 0.9999 |
| 2 | 0.93 | 0.1438 | 0.4485 | 0.6447 | 0.7512 | 0.8873 | 0.9905 | 0.9999 |
| 3 | 0.96 | 0.3122 | 0.0931 | 0.3207 | 0.0890 | 0.2925 | 0.2863 | 0.9999 |
| 4 | 0.99 | 0.2001 | 0.2891 | 0.5241 | 0.4093 | 0.6087 | 0.9999 | 0.9999 |
| 5 | 1.01 | 0.0721 | 0.7052 | 0.8212 | 0.9238 | 0.8582 | 0.9999 | 0.9999 |
| 6 | 1.04 | 0.2590 | 0.1669 | 0.4653 | 0.0564 | 0.2193 | 0.4172 | 0.9999 |
| 7 | 1.26 | -0.1665 | 0.3792 | 0.5800 | 0.4306 | 0.6495 | 0.9999 | 0.9999 |
| 8 | 1.34 | -0.2164 | 0.2507 | 0.5013 | 0.4553 | 0.6756 | 0.8257 | 0.9999 |
| 9 | 1.41 | 0.0415 | 0.8278 | 0.8856 | 0.9351 | 0.8582 | 0.9999 | 0.9999 |
| 10 | 1.45 | -0.3196 | 0.0851 | 0.3131 | 0.0241 | 0.1752 | 0.0582 | 0.5949 |
| 11 | 1.47 | 0.1625 | 0.3909 | 0.5800 | 0.7547 | 0.8728 | 0.9853 | 0.9999 |
| 12 | 1.59 | -0.1670 | 0.3779 | 0.5800 | 0.3401 | 0.5903 | 0.9999 | 0.9999 |
| 13 | 1.69 | 0.3998 | **0.0286** | 0.2254 | **0.0279** | 0.2925 | 0.6792 | 0.9999 |
| 14 | 1.79 | -0.3210 | 0.0837 | 0.3131 | **0.0381** | 0.1752 | 0.3466 | 0.9999 |
| 15 | 1.88 | 0.1133 | 0.5513 | 0.7105 | 0.2808 | 0.5497 | 0.9999 | 0.9999 |
| 16 | 1.92 | 0.2181 | 0.2470 | 0.5013 | 1.0000 | 1.0000 | 0.9999 | 0.9999 |
| 17 | 2.08 | 0.3752 | **0.0410** | 0.2254 | **0.0015** | **0.0429** | **0.0206** | 0.3790 |
| 18 | 2.25 | -0.1664 | 0.3794 | 0.5800 | 0.2998 | 0.5628 | 0.9999 | 0.9999 |
| 19 | 2.30 | -0.1995 | 0.2905 | 0.5241 | 0.0860 | 0.2977 | 0.2494 | 0.9999 |
| 20 | 2.34 | 0.4299 | **0.0177** | 0.2254 | **0.0048** | 0.0717 | **0.0482** | 0.5911 |
| 21 | 2.37 | -0.0302 | 0.8741 | 0.9138 | 0.7689 | 0.8582 | 0.9911 | 0.9999 |
| 22 | 2.38 | 0.1175 | 0.5362 | 0.7047 | 0.5871 | 0.8582 | 0.9436 | 0.9999 |
| 23 | 2.42 | -0.2504 | 0.1820 | 0.4717 | 0.2513 | 0.5372 | 0.6187 | 0.9999 |
| 24 | 2.46 | 0.1819 | 0.3361 | 0.5727 | 0.1985 | 0.4566 | 0.8957 | 0.9999 |
| 25 | 2.55 | -0.2445 | 0.1928 | 0.4717 | 0.5720 | 0.6087 | 0.9999 | 0.9999 |
| 26 | 2.64 | -0.4138 | **0.0230** | 0.2254 | **0.0181** | 0.1511 | 0.0960 | 0.8029 |
| 27 | 2.66 | -0.2445 | 0.1930 | 0.4717 | 0.0164 | 0.1540 | 0.0514 | 0.5911 |
| 28 | 2.71 | -0.2039 | 0.2797 | 0.5241 | 0.5338 | 0.7674 | 0.9999 | 0.9999 |
| 29 | 2.76 | -0.1626 | 0.3908 | 0.5800 | 0.6682 | 0.8582 | 0.9999 | 0.9999 |
| 30 | 2.81 | 0.1686 | 0.3732 | 0.5800 | 0.1577 | 0.4788 | 0.9999 | 0.9999 |
| 31 | 2.87 | 0.2521 | 0.1789 | 0.4717 | 0.9732 | 1.0000 | 0.9999 | 0.9999 |
| 32 | 2.91 | -0.1320 | 0.4868 | 0.6724 | 0.5897 | 0.8221 | 0.9999 | 0.9999 |
| 33 | 2.95 | -0.0664 | 0.7273 | 0.8364 | 0.1096 | 0.2193 | 0.9999 | 0.9999 |
| 34 | 2.99 | 0.2408 | 0.1999 | 0.4717 | 0.0719 | 0.2193 | 0.4299 | 0.9999 |
| 35 | 3.02 | -0.6626 | **0.0001** | **0.0034** | **0.0003** | **0.0309** | 0.0056 | 0.1717 |
| 36 | 3.05 | -0.2302 | 0.2210 | 0.5008 | 0.5653 | 0.8002 | 0.9999 | 0.9999 |
| 37 | 3.09 | -0.0593 | 0.7554 | 0.8476 | 0.9010 | 0.9752 | 0.9999 | 0.9999 |
| 38 | 3.14 | -0.0827 | 0.6640 | 0.7934 | 0.8241 | 0.8582 | 0.9999 | 0.9999 |
| 39 | 3.19 | 0.6173 | **0.0003** | **0.0086** | **0.0055** | 0.0717 | 0.0825 | 0.7590 |
| 40 | 3.22 | 0.3612 | 0.0499 | 0.2259 | 0.1632 | 0.4900 | 0.9999 | 0.9999 |
| 41 | 3.26 | -0.4622 | **0.0101** | 0.1554 | **0.0372** | 0.3040 | 0.2498 | 0.9999 |
| 42 | 3.29 | -0.2803 | 0.1336 | 0.4097 | **0.0412** | 0.2193 | 0.2934 | 0.9999 |
| 43 | 3.34 | -0.0524 | 0.7832 | 0.8681 | 0.7400 | 0.8728 | 0.9999 | 0.9999 |
| 44 | 3.42 | -0.4759 | **0.0079** | 0.1446 | 0.0997 | 0.3206 | 0.3888 | 0.9999 |
| 45 | 3.48 | 0.0211 | 0.9119 | 0.9193 | 0.5338 | 0.7674 | 0.9999 | 0.9999 |
| 46 | 3.53 | -0.2227 | 0.2369 | 0.5008 | 0.4636 | 0.6087 | 0.8246 | 0.9999 |
| 47 | 3.56 | 0.0721 | 0.7051 | 0.8212 | 0.6098 | 0.8582 | 0.9999 | 0.9999 |
| 48 | 3.57 | 0.1745 | 0.3564 | 0.5800 | 0.8955 | 0.9752 | 0.9992 | 0.9999 |
| 49 | 3.60 | -0.3664 | **0.0465** | 0.2254 | 0.1474 | 0.4035 | 0.3784 | 0.9999 |
| 50 | 3.69 | 0.0865 | 0.6493 | 0.7860 | 0.5432 | 0.6087 | 0.9999 | 0.9999 |
| 51 | 3.71 | 0.2215 | 0.2395 | 0.5008 | 1.0000 | 1.0000 | 0.9999 | 0.9999 |
| 52 | 3.73 | -0.2118 | 0.2612 | 0.5113 | 0.0564 | 0.2193 | 0.2579 | 0.9999 |
| 53 | 3.76 | 0.2717 | 0.1464 | 0.4209 | 0.2424 | 0.3460 | 0.5577 | 0.9999 |
| 54 | 3.81 | -0.0626 | 0.7425 | 0.8433 | 0.8084 | 0.9876 | 0.9999 | 0.9999 |
| 55 | 3.85 | 0.0459 | 0.8096 | 0.8762 | 0.2290 | 0.4900 | 0.9999 | 0.9999 |
| 56 | 3.89 | 0.0193 | 0.9193 | 0.9193 | 0.3195 | 0.5764 | 0.9999 | 0.9999 |
| 57 | 3.93 | -0.6596 | **0.0001** | **0.0034** | **0.0003** | **0.0309** | **0.0056** | 0.1717 |
| 58 | 3.98 | 0.1392 | 0.4633 | 0.6557 | 0.6783 | 0.8582 | 0.8618 | 0.9999 |
| 59 | 4.06 | 0.0224 | 0.9067 | 0.9193 | 0.6227 | 0.9876 | 0.9421 | 0.9999 |
| 60 | 4.12 | -0.1592 | 0.4009 | 0.5854 | 0.4470 | 0.4035 | 0.8368 | 0.9999 |
| 61 | 4.18 | -0.0951 | 0.6173 | 0.7598 | 0.2743 | 0.5487 | 0.9999 | 0.9999 |
| 62 | 4.21 | 0.3685 | **0.0451** | 0.2254 | 0.1248 | 0.3377 | 0.6209 | 0.9999 |
| 63 | 4.26 | -0.1034 | 0.5866 | 0.7392 | 0.6016 | 0.9126 | 0.9301 | 0.9999 |
| 64 | 4.32 | 0.3587 | 0.0516 | 0.2259 | 0.7089 | 0.8582 | 0.9999 | 0.9999 |
| 65 | 4.41 | -0.3373 | 0.0684 | 0.2734 | 0.0381 | 0.1752 | 0.2319 | 0.9999 |
| 66 | 4.44 | -0.1211 | 0.5239 | 0.6986 | 0.9011 | 0.8582 | 0.9999 | 0.9999 |
| 67 | 4.52 | 0.2766 | 0.1390 | 0.4125 | 0.5837 | 1.0000 | 0.9999 | 0.9999 |
| 68 | 4.58 | 0.0321 | 0.8662 | 0.9138 | 0.3106 | 0.5764 | 0.7662 | 0.9999 |
| 69 | 4.65 | -0.4035 | **0.0270** | 0.2254 | **0.0042** | 0.0717 | **0.0185** | 0.3790 |
| 70 | 5.88 | 0.3112 | 0.0941 | 0.3207 | 0.3615 | 0.6087 | 0.9999 | 0.9999 |
| 71 | 5.92 | 0.3930 | **0.0317** | 0.2254 | 0.4068 | 0.6237 | 0.9999 | 0.9999 |
| 72 | 5.97 | -0.1233 | 0.5164 | 0.6986 | 0.4068 | 0.6237 | 0.9999 | 0.9999 |
| 73 | 6.09 | -0.3704 | 0.0439 | 0.2254 | **0.0127** | 0.1540 | 0.2082 | 0.9999 |
| 74 | 6.52 | 0.1311 | 0.4897 | 0.6724 | 0.5127 | 0.5903 | 0.9999 | 0.9999 |
| 75 | 6.61 | -0.0224 | 0.9066 | 0.9193 | 0.9669 | 1.0000 | 0.9999 | 0.9999 |
| 76 | 6.79 | -0.5139 | **0.0037** | 0.0845 | **0.0059** | 0.1511 | 0.1148 | 0.8801 |
| 77 | 6.88 | 0.1869 | 0.3228 | 0.5603 | 0.0971 | 0.2977 | 0.7255 | 0.9999 |
| 78 | 6.99 | 0.0945 | 0.6194 | 0.7598 | 0.4222 | 0.5628 | 0.8710 | 0.9999 |
| 79 | 7.17 | 0.1904 | 0.3135 | 0.5547 | 0.1960 | 0.3460 | 0.4529 | 0.9999 |
| 80 | 7.20 | 0.2281 | 0.2254 | 0.5008 | 0.9010 | 0.9752 | 0.9999 | 0.9999 |
| 81 | 7.31 | 0.2946 | 0.1141 | 0.3619 | 0.0620 | 0.2193 | 0.9999 | 0.9999 |
| 82 | 7.36 | 0.3782 | **0.0393** | 0.2254 | **0.0131** | 0.1540 | 0.2026 | 0.9999 |
| 83 | 7.41 | 0.2432 | 0.1953 | 0.4717 | 0.0850 | 0.3040 | 0.9999 | 0.9999 |
| 84 | 7.73 | 0.2243 | 0.2334 | 0.5008 | 0.9600 | 0.9741 | 0.9999 | 0.9999 |
| 85 | 7.90 | 0.3842 | **0.0361** | 0.2254 | 0.1284 | 0.4035 | 0.9999 | 0.9999 |
| 86 | 7.96 | -0.3027 | 0.1039 | 0.3415 | **0.0344** | 0.1752 | 0.1367 | 0.8983 |
| 87 | 8.17 | 0.3428 | 0.0637 | 0.2663 | **0.0019** | 0.0429 | 0.0056 | 0.1717 |
| 88 | 8.23 | -0.3662 | **0.0466** | 0.2254 | **0.0246** | 0.1540 | 0.1765 | 0.9999 |
| 89 | 8.35 | -0.4065 | **0.0258** | 0.2254 | **0.0102** | 0.1472 | 0.1270 | 0.8983 |
| 90 | 8.41 | -0.1701 | 0.3687 | 0.5800 | **0.0128** | 0.1472 | **0.0453** | 0.5911 |
| 91 | 8.60 | -0.2078 | 0.2705 | 0.5185 | **0.0263** | 0.1752 | 0.2382 | 0.9999 |
| 92 | 8.93 | 0.0472 | 0.8045 | 0.8762 | 0.7716 | 0.8873 | 0.9999 | 0.9999 |

q-value is based on Benjamini-Hochberg correction.
