# Supplementary material for: Metabolic readouts of tumor instructed normal tissues (TINT) identify aggressive prostate cancer subgroups for tailored therapy
Source: Front Mol Biosci. 2025 Apr 7;12:1426949. doi: 10.3389/fmolb.2025.1426949 (PMC12009692; doi:10.3389/fmolb.2025.1426949)
Supplement: Supplementary file 9 [file Table3.docx]

**Table S3. Comparison of benign samples accompanying tumors with ISUP 1+2 and ISUP 3+4 from PC patients with unifocal tumors only (n = 27) for all integrated variables.**

| **Nr** | **Chemical shift (ppm)** | **Correlation with ISUP values** | | | **B ISUP 1+2 *vs* B ISUP 3+4** | | **Post-hoc analysis B ISUP 1+2 *vs***  **B ISUP 3+4** | |
| --- | --- | --- | --- | --- | --- | --- | --- | --- |
|  |  | **coefficient** | ***p*-value** | **q value*** | ***p*-value** | **q value*** | ***p*-value** | **q value*** |
| 1 | 0.87 | -0.0497 | 0.8057 | 0.9392 | 0.8034 | 0.9476 | >0.9999 | 0.9999 |
| 2 | 0.93 | 0.1557 | 0.4382 | 0.9375 | 0.7225 | 0.9476 | 0.9808 | 0.9999 |
| 3 | 0.96 | -0.1352 | 0.5013 | 0.9392 | 0.4799 | 0.9476 | >0.9999 | 0.9999 |
| 4 | 0.99 | -0.0490 | 0.8081 | 0.9392 | 0.9339 | 1.0000 | >0.9999 | 0.9999 |
| 5 | 1.01 | 0.1129 | 0.5751 | 0.9392 | 0.6381 | 0.9476 | >0.9999 | 0.9999 |
| 6 | 1.04 | -0.0252 | 0.9008 | 0.9392 | 0.8034 | 0.9476 | >0.9999 | 0.9999 |
| 7 | 1.26 | -0.1073 | 0.5941 | 0.9392 | 0.4224 | 0.8832 | >0.9999 | 0.9999 |
| 8 | 1.34 | -0.2586 | 0.1929 | 0.8067 | 0.1752 | 0.7425 | 0.5332 | 0.9999 |
| 9 | 1.41 | 0.4869 | **0.0100** | 0.2300 | 0.0807 | 0.6805 | **0.0280** | 0.9999 |
| 10 | 1.45 | -0.0274 | 0.8923 | 0.9392 | 0.8034 | 0.9476 | 0.9979 | 0.9999 |
| 11 | 1.47 | 0.0678 | 0.7369 | 0.9392 | 0.8900 | 1.0000 | 0.9981 | 0.9999 |
| 12 | 1.59 | -0.1015 | 0.6145 | 0.9392 | 0.1026 | 0.7133 | 0.5922 | 0.9999 |
| 13 | 1.69 | 0.0976 | 0.6282 | 0.9392 | 0.7191 | 0.9476 | >0.9999 | 0.9999 |
| 14 | 1.79 | 0.1395 | 0.4877 | 0.9392 | 0.6782 | 0.9476 | >0.9999 | 0.9999 |
| 15 | 1.88 | 0.3216 | 0.1019 | 0.7430 | 0.0788 | 0.6805 | 0.7336 | 0.9999 |
| 16 | 1.92 | -0.1745 | 0.3839 | 0.9056 | 0.7191 | 0.9476 | >0.9999 | 0.9999 |
| 17 | 2.08 | 0.1816 | 0.3645 | 0.9056 | 0.1426 | 0.7425 | >0.9999 | 0.9999 |
| 18 | 2.25 | -0.0964 | 0.6324 | 0.9392 | 0.8034 | 0.9476 | >0.9999 | 0.9999 |
| 19 | 2.30 | 0.1855 | 0.3543 | 0.9056 | 0.3911 | 0.8832 | 0.8799 | 0.9999 |
| 20 | 2.34 | 0.1667 | 0.4058 | 0.9333 | 0.4224 | 0.8832 | >0.9999 | 0.9999 |
| 21 | 2.37 | -0.0379 | 0.8511 | 0.9392 | 0.5992 | 0.9476 | 0.9799 | 0.9999 |
| 22 | 2.38 | -0.0316 | 0.8755 | 0.9392 | 0.5992 | 0.9476 | 0.9497 | 0.9999 |
| 23 | 2.42 | 0.1113 | 0.5806 | 0.9392 | 0.7609 | 0.9476 | 0.9996 | 0.9999 |
| 24 | 2.46 | 0.2086 | 0.2963 | 0.8793 | 0.3060 | 0.8832 | 0.7386 | 0.9999 |
| 25 | 2.55 | 0.3664 | 0.0601 | 0.6379 | **0.0261** | 0.6805 | >0.9999 | 0.9999 |
| 26 | 2.64 | -0.1017 | 0.6137 | 0.9392 | 0.7609 | 0.9476 | >0.9999 | 0.9999 |
| 27 | 2.66 | 0.0430 | 0.8314 | 0.9392 | 0.6381 | 0.9476 | 0.9634 | 0.9999 |
| 28 | 2.71 | 0.3023 | 0.1254 | 0.7430 | 0.2806 | 0.8832 | >0.9999 | 0.9999 |
| 29 | 2.76 | -0.1405 | 0.4845 | 0.9392 | 0.3944 | 0.8832 | >0.9999 | 0.9999 |
| 30 | 2.81 | -0.0458 | 0.8207 | 0.9392 | 1.0000 | 1.0000 | >0.9999 | 0.9999 |
| 31 | 2.87 | 0.1571 | 0.4339 | 0.9375 | 0.8034 | 0.9476 | >0.9999 | 0.9999 |
| 32 | 2.91 | 0.2668 | 0.1786 | 0.8067 | 0.0721 | 0.6805 | 0.6216 | 0.9999 |
| 33 | 2.95 | -0.0902 | 0.6548 | 0.9392 | 0.4215 | 0.8832 | >0.9999 | 0.9999 |
| 34 | 2.99 | 0.3654 | 0.0609 | 0.6379 | 0.1012 | 0.7133 | 0.2024 | 0.9999 |
| 35 | 3.02 | -0.3229 | 0.1005 | 0.7430 | 0.1498 | 0.7425 | >0.9999 | 0.9999 |
| 36 | 3.05 | 0.5122 | **0.0063** | 0.2300 | 0.9643 | 1.0000 | >0.9999 | 0.9999 |
| 37 | 3.09 | 0.1968 | 0.3252 | 0.9056 | 0.4892 | 0.9476 | >0.9999 | 0.9999 |
| 38 | 3.14 | -0.1314 | 0.5137 | 0.9392 | 0.9779 | 1.0000 | >0.9999 | 0.9999 |
| 39 | 3.19 | 0.1859 | 0.3533 | 0.9056 | 0.8900 | 1.0000 | >0.9999 | 0.9999 |
| 40 | 3.22 | -0.0744 | 0.7122 | 0.9392 | 0.6077 | 0.9476 | >0.9999 | 0.9999 |
| 41 | 3.26 | -0.0471 | 0.8156 | 0.9392 | 0.5613 | 0.9476 | 0.9737 | 0.9999 |
| 42 | 3.29 | 0.0511 | 0.8002 | 0.9392 | 0.9779 | 1.0000 | >0.9999 | 0.9999 |
| 43 | 3.34 | 0.0613 | 0.7615 | 0.9392 | 0.3613 | 0.8832 | >0.9999 | 0.9999 |
| 44 | 3.42 | -0.2966 | 0.1330 | 0.7430 | 0.1662 | 0.7425 | >0.9999 | 0.9999 |
| 45 | 3.48 | 0.0983 | 0.6257 | 0.9392 | 0.6782 | 0.9476 | >0.9999 | 0.9999 |
| 46 | 3.53 | 0.3857 | **0.0469** | 0.6379 | 0.0721 | 0.6805 | 0.5641 | 0.9999 |
| 47 | 3.56 | -0.0282 | 0.8891 | 0.9392 | 0.9810 | 1.0000 | 0.5641 | 0.9999 |
| 48 | 3.57 | -0.0661 | 0.7432 | 0.9392 | 0.9339 | 1.0000 | 0.9656 | 0.9999 |
| 49 | 3.60 | 0.2991 | 0.1296 | 0.7430 | 0.2707 | 0.8832 | 0.7449 | 0.9999 |
| 50 | 3.69 | -0.0559 | 0.7819 | 0.9392 | 0.6782 | 0.9476 | >0.9999 | 0.9999 |
| 51 | 3.71 | 0.0849 | 0.6739 | 0.9392 | 0.9497 | 1.0000 | >0.9999 | 0.9999 |
| 52 | 3.73 | -0.3169 | 0.1073 | 0.7430 | 0.0714 | 0.6805 | >0.9999 | 0.9999 |
| 53 | 3.76 | 0.2208 | 0.2683 | 0.8512 | 0.7609 | 0.9476 | 0.8702 | 0.9999 |
| 54 | 3.81 | -0.2749 | 0.1652 | 0.8067 | 0.0638 | 0.6805 | 0.3331 | 0.9999 |
| 55 | 3.85 | -0.1927 | 0.3356 | 0.9056 | 0.2806 | 0.8832 | >0.9999 | 0.9999 |
| 56 | 3.89 | -0.2731 | 0.1681 | 0.8067 | 0.0814 | 0.6805 | 0.4432 | 0.9999 |
| 57 | 3.93 | -0.2935 | 0.1373 | 0.7430 | 0.2132 | 0.8527 | >0.9999 | 0.9999 |
| 58 | 3.98 | 0.1817 | 0.3645 | 0.9056 | 0.3911 | 0.8832 | 0.7830 | 0.9999 |
| 59 | 4.06 | 0.3724 | 0.0557 | 0.6379 | 0.1426 | 0.7425 | 0.5680 | 0.9999 |
| 60 | 4.12 | -0.2164 | 0.2783 | 0.8535 | 0.1494 | 0.7425 | 0.5468 | 0.9999 |
| 61 | 4.18 | 0.0146 | 0.9425 | 0.9529 | 0.3908 | 0.8832 | >0.9999 | 0.9999 |
| 62 | 4.21 | -0.2217 | 0.2663 | 0.8512 | 0.1085 | 0.7133 | 0.6655 | 0.9999 |
| 63 | 4.26 | -0.0364 | 0.8570 | 0.9392 | 0.5992 | 0.9476 | 0.8919 | 0.9999 |
| 64 | 4.32 | -0.1617 | 0.4204 | 0.9375 | 0.3911 | 0.8832 | >0.9999 | 0.9999 |
| 65 | 4.41 | -0.2340 | 0.2400 | 0.8492 | 0.2567 | 0.8832 | >0.9999 | 0.9999 |
| 66 | 4.44 | 0.3634 | 0.0624 | 0.6379 | 0.5992 | 0.9476 | >0.9999 | 0.9999 |
| 67 | 4.52 | -0.0557 | 0.7827 | 0.9392 | 0.8465 | 0.9857 | >0.9999 | 0.9999 |
| 68 | 4.58 | 0.0292 | 0.8851 | 0.9392 | 0.4551 | 0.9304 | 0.9814 | 0.9999 |
| 69 | 4.65 | -0.2241 | 0.2612 | 0.8512 | 0.1776 | 0.7425 | >0.9999 | 0.9999 |
| 70 | 5.88 | -0.1757 | 0.3807 | 0.9056 | 0.1752 | 0.7425 | >0.9999 | 0.9999 |
| 71 | 5.92 | -0.0849 | 0.6739 | 0.9392 | 0.5209 | 0.9476 | >0.9999 | 0.9999 |
| 72 | 5.97 | 0.1496 | 0.4563 | 0.9392 | 0.6376 | 0.9476 | >0.9999 | 0.9999 |
| 73 | 6.09 | -0.0232 | 0.9086 | 0.9392 | 0.7955 | 0.9476 | >0.9999 | 0.9999 |
| 74 | 6.52 | 0.0409 | 0.8394 | 0.9392 | 0.7336 | 0.9476 | >0.9999 | 0.9999 |
| 75 | 6.61 | 0.5620 | **0.0023** | 0.2116 | **0.0380** | 0.6805 | 0.2794 | 0.9999 |
| 76 | 6.79 | -0.2389 | 0.2301 | 0.8468 | 0.2342 | 0.8832 | >0.9999 | 0.9999 |
| 77 | 6.88 | -0.0300 | 0.8821 | 0.9392 | 0.9339 | 1.0000 | >0.9999 | 0.9999 |
| 78 | 6.99 | 0.5000 | **0.0079** | 0.2300 | 0.0721 | 0.6805 | >0.9999 | 0.9999 |
| 79 | 7.17 | 0.1166 | 0.5626 | 0.9392 | 0.4224 | 0.8832 | 0.9539 | 0.9999 |
| 80 | 7.20 | 0.1104 | 0.5834 | 0.9392 | 0.3613 | 0.8832 | >0.9999 | 0.9999 |
| 81 | 7.31 | -0.2441 | 0.2199 | 0.8468 | 0.3329 | 0.8832 | >0.9999 | 0.9999 |
| 82 | 7.36 | 0.2609 | 0.1887 | 0.8067 | 0.3329 | 0.8832 | >0.9999 | 0.9999 |
| 83 | 7.41 | -0.0582 | 0.7730 | 0.9392 | 0.8034 | 0.9476 | >0.9999 | 0.9999 |
| 84 | 7.73 | 0.2434 | 0.2211 | 0.8468 | 0.3060 | 0.8832 | 0.9312 | 0.9999 |
| 85 | 7.90 | -0.0199 | 0.9215 | 0.9420 | 0.5619 | 0.9476 | >0.9999 | 0.9999 |
| 86 | 7.96 | -0.1395 | 0.4876 | 0.9392 | 0.5992 | 0.9476 | >0.9999 | 0.9999 |
| 87 | 8.17 | 0.0254 | 0.9000 | 0.9392 | 0.6381 | 0.9476 | >0.9999 | 0.9999 |
| 88 | 8.23 | -0.0325 | 0.8722 | 0.9392 | 1.0000 | 1.0000 | >0.9999 | 0.9999 |
| 89 | 8.35 | 0.0103 | 0.9592 | 0.9592 | 0.9310 | 1.0000 | 0.9996 | 0.9999 |
| 90 | 8.41 | 0.1262 | 0.5304 | 0.9392 | 0.7191 | 0.9476 | 0.9919 | 0.9999 |
| 91 | 8.60 | -0.3530 | 0.0709 | 0.6523 | **0.0412** | 0.6805 | 0.1536 | 0.9999 |
| 92 | 8.93 | -0.0376 | 0.8524 | 0.9392 | 0.3613 | 0.8832 | >0.9999 | 0.9999 |

q-value is based on Benjamini-Hochberg correction.
