# Supplementary material for: Metabolic readouts of tumor instructed normal tissues (TINT) identify aggressive prostate cancer subgroups for tailored therapy
Source: Front Mol Biosci. 2025 Apr 7;12:1426949. doi: 10.3389/fmolb.2025.1426949 (PMC12009692; doi:10.3389/fmolb.2025.1426949)
Supplement: Supplementary file 10 [file Table4.docx]

**Table S4. Comparison of tumor samples with ISUP 1+2 and ISUP 3+4 from PC patients with unifocal and selected multifocal tumors (n = 48) for all integrated variables.**

| **Nr** | **Chemical shift (ppm)** | **Correlation with ISUP values** | | | **PC ISUP 1+2 *vs* PC ISUP 3+4** | | **Post-hoc analysis PC ISUP 1+2 *vs***  **PC ISUP 3+4** | |
| --- | --- | --- | --- | --- | --- | --- | --- | --- |
|  |  | **coefficient** | ***p*-value** | **q value*** | ***p*-value** | **q value*** | ***p*-value** | **q value*** |
| 1 | 0.87 | 0.0469 | 0.7516 | 0.8537 | **0.0097** | 0.0524 | 0.0644 | 0.3385 |
| 2 | 0.93 | 0.0806 | 0.5860 | 0.7814 | 0.6527 | 0.7699 | 0.9747 | 0.9999 |
| 3 | 0.96 | -0.2065 | 0.1591 | 0.3727 | 0.4903 | 0.6699 | 0.9999 | 0.9999 |
| 4 | 0.99 | 0.0975 | 0.5099 | 0.6959 | 0.9212 | 0.9313 | 0.9997 | 0.9999 |
| 5 | 1.01 | 0.0727 | 0.6232 | 0.7963 | 0.6046 | 0.7517 | 0.9999 | 0.9999 |
| 6 | 1.04 | 0.1292 | 0.3814 | 0.5899 | 0.3885 | 0.6059 | 0.9999 | 0.9999 |
| 7 | 1.26 | -0.0754 | 0.6105 | 0.7911 | 0.1885 | 0.4110 | 0.8061 | 0.9999 |
| 8 | 1.34 | -0.3374 | **0.0190** | 0.1165 | **0.0343** | 0.1434 | 0.1557 | 0.6511 |
| 9 | 1.41 | 0.0275 | 0.8528 | 0.9230 | 0.8631 | 0.9127 | 0.9999 | 0.9999 |
| 10 | 1.45 | -0.2867 | **0.0482** | 0.2015 | **0.0089** | 0.0513 | **0.0429** | 0.2519 |
| 11 | 1.47 | 0.0525 | 0.7230 | 0.8315 | 0.6508 | 0.7699 | 0.9999 | 0.9999 |
| 12 | 1.59 | -0.0760 | 0.6075 | 0.7911 | 0.0702 | 0.2084 | 0.6997 | 0.9999 |
| 13 | 1.69 | -0.0327 | 0.8252 | 0.9038 | 0.0669 | 0.2084 | 0.3988 | 0.9999 |
| 14 | 1.79 | -0.2032 | 0.1661 | 0.3727 | **0.0395** | 0.1581 | 0.4334 | 0.9999 |
| 15 | 1.88 | 0.0361 | 0.8078 | 0.8953 | 0.9313 | 0.9313 | 0.9999 | 0.9999 |
| 16 | 1.92 | 0.1686 | 0.2521 | 0.4817 | 0.2716 | 0.5014 | 0.9999 | 0.9999 |
| 17 | 2.08 | 0.4118 | **0.0036** | **0.0380** | **<0.0001** | **0.0020** | **0.0026** | 0.0598 |
| 18 | 2.25 | -0.1283 | 0.3847 | 0.5899 | 0.6200 | 0.7606 | 0.9999 | 0.9999 |
| 19 | 2.30 | -0.2041 | 0.1641 | 0.3727 | **0.0145** | 0.0704 | 0.1288 | 0.5643 |
| 20 | 2.34 | 0.4360 | **0.0020** | **0.0299** | **0.0006** | **0.0114** | **0.0015** | 0.0598 |
| 21 | 2.37 | 0.0024 | 0.9872 | 0.9980 | 0.7336 | 0.8436 | 0.9861 | 0.9999 |
| 22 | 2.38 | 0.2112 | 0.1496 | 0.3727 | 0.3228 | 0.5604 | 0.7426 | 0.9999 |
| 23 | 2.42 | -0.1363 | 0.3556 | 0.5839 | 0.4087 | 0.6157 | 0.9999 | 0.9999 |
| 24 | 2.46 | 0.0132 | 0.9289 | 0.9602 | 0.0638 | 0.2084 | 0.6461 | 0.9999 |
| 25 | 2.55 | -0.2671 | 0.0665 | 0.2447 | 0.1905 | 0.4110 | 0.7076 | 0.9999 |
| 26 | 2.64 | -0.2047 | 0.1628 | 0.3727 | **0.0177** | 0.0815 | 0.0699 | 0.3385 |
| 27 | 2.66 | -0.0613 | 0.6788 | 0.8315 | 0.1128 | 0.2883 | 0.6677 | 0.9999 |
| 28 | 2.71 | -0.2001 | 0.1727 | 0.3759 | 0.1677 | 0.3857 | 0.9999 | 0.9999 |
| 29 | 2.76 | -0.0686 | 0.6431 | 0.7995 | 0.4253 | 0.6157 | 0.9999 | 0.9999 |
| 30 | 2.81 | 0.1549 | 0.2933 | 0.5291 | 0.0638 | 0.2084 | 0.9999 | 0.9999 |
| 31 | 2.87 | 0.2229 | 0.1277 | 0.3727 | 0.8631 | 0.9127 | 0.9999 | 0.9999 |
| 32 | 2.91 | -0.2725 | 0.0609 | 0.2336 | 0.8631 | 0.9127 | 0.9999 | 0.9999 |
| 33 | 2.95 | -0.1778 | 0.2266 | 0.4533 | **0.0236** | 0.1034 | 0.103 | 0.4738 |
| 34 | 2.99 | 0.1582 | 0.2830 | 0.5207 | 0.0969 | 0.2623 | 0.8914 | 0.9999 |
| 35 | 3.02 | -0.5162 | **0.0002** | **0.0053** | **0.0004** | **0.0095** | **0.0068** | 0.1043 |
| 36 | 3.05 | -0.1218 | 0.4096 | 0.5982 | 0.8365 | 0.9127 | 0.9999 | 0.9999 |
| 37 | 3.09 | 0.0562 | 0.7042 | 0.8315 | 0.5319 | 0.6990 | 0.9999 | 0.9999 |
| 38 | 3.14 | -0.1098 | 0.4575 | 0.6475 | 0.6820 | 0.7942 | 0.9999 | 0.9999 |
| 39 | 3.19 | 0.5463 | **0.0001** | **0.0033** | **0.0008** | **0.0128** | **0.013** | 0.1495 |
| 40 | 3.22 | 0.4109 | **0.0037** | **0.0380** | **0.0473** | 0.1742 | 0.4893 | 0.9999 |
| 41 | 3.26 | -0.4006 | **0.0048** | 0.0440 | 0.0702 | 0.2084 | 0.3095 | 0.9999 |
| 42 | 3.29 | -0.1326 | 0.3688 | 0.5849 | 0.2032 | 0.4110 | 0.6356 | 0.9999 |
| 43 | 3.34 | -0.1987 | 0.1757 | 0.3759 | 0.5319 | 0.6990 | 0.9999 | 0.9999 |
| 44 | 3.42 | -0.4969 | **0.0003** | 0.0075 | **0.0420** | 0.1612 | 0.2876 | 0.9999 |
| 45 | 3.48 | -0.0196 | 0.8947 | 0.9430 | 0.2444 | 0.4684 | 0.9999 | 0.9999 |
| 46 | 3.53 | -0.1238 | 0.4019 | 0.5964 | 0.5787 | 0.7293 | 0.9282 | 0.9999 |
| 47 | 3.56 | 0.1394 | 0.3448 | 0.5839 | 0.3479 | 0.5713 | 0.9999 | 0.9999 |
| 48 | 3.57 | 0.0533 | 0.7189 | 0.8315 | 0.8759 | 0.9157 | 0.9999 | 0.9999 |
| 49 | 3.60 | -0.1913 | 0.1929 | 0.4033 | 0.2725 | 0.5014 | 0.6695 | 0.9999 |
| 50 | 3.69 | 0.2354 | 0.1072 | 0.3289 | 0.0550 | 0.1947 | 0.3756 | 0.9999 |
| 51 | 3.71 | 0.1441 | 0.3285 | 0.5702 | 0.4250 | 0.6157 | 0.9999 | 0.9999 |
| 52 | 3.73 | -0.2092 | 0.1536 | 0.3727 | **0.0110** | 0.0561 | 0.0673 | 0.3385 |
| 53 | 3.76 | 0.2123 | 0.1474 | 0.3727 | 0.1326 | 0.3211 | 0.3945 | 0.9999 |
| 54 | 3.81 | -0.1183 | 0.4234 | 0.6086 | 0.4283 | 0.6157 | 0.9999 | 0.9999 |
| 55 | 3.85 | -0.1346 | 0.3618 | 0.5839 | 0.5752 | 0.7293 | 0.9999 | 0.9999 |
| 56 | 3.89 | 0.0053 | 0.9713 | 0.9929 | 0.3653 | 0.5794 | 0.9999 | 0.9999 |
| 57 | 3.93 | -0.5409 | **0.0001** | **0.0033** | **0.0001** | **0.0021** | **0.0026** | 0.0598 |
| 58 | 3.98 | 0.1890 | 0.1981 | 0.4051 | 0.4506 | 0.6377 | 0.5168 | 0.9999 |
| 59 | 4.06 | 0.0532 | 0.7195 | 0.8315 | 0.4951 | 0.6699 | 0.8902 | 0.9999 |
| 60 | 4.12 | -0.2406 | 0.0995 | 0.3156 | 0.1571 | 0.3706 | 0.4368 | 0.9999 |
| 61 | 4.18 | -0.0003 | 0.9984 | 0.9984 | 0.2055 | 0.4110 | 0.8569 | 0.9999 |
| 62 | 4.21 | 0.3835 | **0.0071** | 0.0547 | 0.2034 | 0.4110 | 0.9999 | 0.9999 |
| 63 | 4.26 | -0.0964 | 0.5144 | 0.6959 | 0.6317 | 0.7647 | 0.9999 | 0.9999 |
| 64 | 4.32 | 0.3741 | **0.0088** | 0.0623 | 0.3540 | 0.5713 | 0.9999 | 0.9999 |
| 65 | 4.41 | -0.2415 | 0.0982 | 0.3156 | **0.0085** | 0.0513 | **0.0438** | 0.2519 |
| 66 | 4.44 | -0.1347 | 0.3615 | 0.5839 | 0.4619 | 0.6439 | 0.9999 | 0.9999 |
| 67 | 4.52 | 0.1737 | 0.2376 | 0.4651 | 0.9142 | 0.9313 | 0.9999 | 0.9999 |
| 68 | 4.58 | -0.1670 | 0.2565 | 0.4817 | 0.0912 | 0.2543 | 0.2968 | 0.9999 |
| 69 | 4.65 | -0.4295 | **0.0023** | **0.0304** | **<0.0001** | **0.0020** | **0.0001** | **0.0092** |
| 70 | 5.88 | 0.2915 | **0.0444** | 0.1945 | 0.3540 | 0.5713 | 0.9999 | 0.9999 |
| 71 | 5.92 | 0.3906 | 0.0061 | 0.0507 | 0.2034 | 0.4110 | 0.9999 | 0.9999 |
| 72 | 5.97 | -0.1248 | 0.3982 | 0.5964 | 0.2274 | 0.4450 | 0.9111 | 0.9999 |
| 73 | 6.09 | -0.3246 | **0.0244** | 0.1319 | **0.0031** | **0.0286** | **0.0381** | 0.2519 |
| 74 | 6.52 | 0.0693 | 0.6398 | 0.7995 | 0.4251 | 0.6157 | 0.9999 | 0.9999 |
| 75 | 6.61 | -0.0182 | 0.9020 | 0.9430 | 0.9313 | 0.9313 | 0.9999 | 0.9999 |
| 76 | 6.79 | -0.4529 | **0.0012** | **0.0226** | **0.0051** | **0.0359** | **0.0393** | 0.2519 |
| 77 | 6.88 | 0.1455 | 0.3238 | 0.5702 | 0.3320 | 0.5657 | 0.9999 | 0.9999 |
| 78 | 6.99 | 0.0248 | 0.8671 | 0.9276 | 0.7796 | 0.8855 | 0.9909 | 0.9999 |
| 79 | 7.17 | 0.2153 | 0.1417 | 0.3727 | 0.2808 | 0.5065 | 0.8851 | 0.9999 |
| 80 | 7.20 | -0.1055 | 0.4752 | 0.6625 | 0.5678 | 0.7293 | 0.9999 | 0.9999 |
| 81 | 7.31 | 0.2590 | 0.0755 | 0.2597 | 0.0846 | 0.2432 | 0.4815 | 0.9999 |
| 82 | 7.36 | 0.2162 | 0.1399 | 0.3727 | 0.1200 | 0.2984 | 0.9505 | 0.9999 |
| 83 | 7.41 | 0.2184 | 0.1358 | 0.3727 | 0.1040 | 0.2733 | 0.9999 | 0.9999 |
| 84 | 7.73 | 0.0554 | 0.7082 | 0.8315 | 0.8509 | 0.9127 | 0.9977 | 0.9999 |
| 85 | 7.90 | 0.3191 | **0.0270** | 0.1382 | 0.3008 | 0.5322 | 0.9999 | 0.9999 |
| 86 | 7.96 | -0.2584 | 0.0762 | 0.2597 | **0.0036** | **0.0286** | **0.0097** | 0.1275 |
| 87 | 8.17 | 0.3382 | **0.0187** | 0.1165 | **0.0034** | **0.0286** | **0.0396** | 0.2519 |
| 88 | 8.23 | -0.3155 | **0.0289** | 0.1401 | **0.0070** | **0.0463** | **0.0410** | 0.2519 |
| 89 | 8.35 | -0.3074 | **0.0335** | 0.1542 | **0.0037** | **0.0286** | **0.0312** | 0.2519 |
| 90 | 8.41 | -0.2772 | **0.0564** | 0.2257 | **0.0032** | **0.0286** | **0.0061** | 0.1043 |
| 91 | 8.60 | -0.3254 | **0.0240** | 0.1319 | **0.0027** | **0.0286** | **0.0184** | 0.1881 |
| 92 | 8.93 | 0.0379 | 0.7981 | 0.8953 | 0.8631 | 0.9127 | 0.9999 | 0.9999 |

q-value is based on Benjamini-Hochberg correction.
