# Supplementary material for: Metabolic readouts of tumor instructed normal tissues (TINT) identify aggressive prostate cancer subgroups for tailored therapy
Source: Front Mol Biosci. 2025 Apr 7;12:1426949. doi: 10.3389/fmolb.2025.1426949 (PMC12009692; doi:10.3389/fmolb.2025.1426949)
Supplement: Supplementary file 11 [file Table6.docx]

**Table S6. Comparison of benign samples based on the distance from the closest tumor from PC patients with unifocal and selected multifocal tumors (n = 40) for all integrated variables.**

| **Nr** | **Chemical shift (ppm)** | **Correlation with distance from the tumor** | | | **0.5 cm ≤ d < 1 cm**  ***vs* d ≥ 1 cm** | | **Post-hoc analysis 0.5 cm ≤ d < 1 cm**  ***vs* d ≥ 1 cm** | |
| --- | --- | --- | --- | --- | --- | --- | --- | --- |
|  |  | **coefficient** | ***p*-value** | **q value*** | ***p*-value** | **q value*** | ***p*-value** | **q value*** |
| 1 | 0.87 | 0.1623 | 0.3171 | 0.8321 | 0.89228 | 0.977259 | 0.9999 | 0.9999 |
| 2 | 0.93 | -0.06184 | 0.7046 | 0.9269 | 0.424758 | 0.685557 | 0.6634 | 0.9999 |
| 3 | 0.96 | -0.08118 | 0.6185 | 0.9178 | 0.870909 | 0.977259 | 0.9999 | 0.9999 |
| 4 | 0.99 | -0.09529 | 0.5586 | 0.8975 | 0.531032 | 0.737966 | 0.7972 | 0.9999 |
| 5 | 1.01 | -0.02488 | 0.8789 | 0.9416 | 0.401131 | 0.685557 | 0.9999 | 0.9999 |
| 6 | 1.04 | -0.03086 | 0.8501 | 0.9416 | 0.569517 | 0.737966 | 0.9999 | 0.9999 |
| 7 | 1.26 | 0.1711 | 0.2911 | 0.8321 | 0.386114 | 0.685557 | 0.9366 | 0.9999 |
| 8 | 1.34 | -0.2825 | 0.0773 | 0.5727 | 0.220978 | 0.613459 | 0.3692 | 0.9999 |
| 9 | 1.41 | 0.09897 | 0.5435 | 0.8975 | 0.89223 | 0.977259 | 0.9999 | 0.9999 |
| 10 | 1.45 | -0.1569 | 0.3335 | 0.8321 | 0.061652 | 0.365359 | 0.2686 | 0.9999 |
| 11 | 1.47 | 0.004134 | 0.9798 | 0.9977 | 0.316292 | 0.683459 | 0.9999 | 0.9999 |
| 12 | 1.59 | 0.1128 | 0.4884 | 0.8975 | 0.481319 | 0.729851 | 0.6872 | 0.9999 |
| 13 | 1.69 | 0.02847 | 0.8616 | 0.9416 | 0.233381 | 0.613459 | 0.9999 | 0.9999 |
| 14 | 1.79 | -0.1917 | 0.2361 | 0.8321 | 0.067512 | 0.365359 | 0.0976 | 0.9999 |
| 15 | 1.88 | -0.3308 | **0.0371** | 0.5727 | 0.065518 | 0.365359 | 0.2074 | 0.9999 |
| 16 | 1.92 | 0.06762 | 0.6785 | 0.9269 | 0.266808 | 0.632854 | 0.3445 | 0.9999 |
| 17 | 2.08 | 0.09122 | 0.5756 | 0.8975 | 0.12264 | 0.40296 | 0.6669 | 0.9999 |
| 18 | 2.25 | 0.03173 | 0.8459 | 0.9416 | 0.12264 | 0.40296 | 0.2933 | 0.9999 |
| 19 | 2.30 | -0.1769 | 0.2749 | 0.8321 | 0.104562 | 0.400821 | 0.2626 | 0.9999 |
| 20 | 2.34 | 0.1982 | 0.2202 | 0.8321 | **0.042225** | 0.323725 | 0.2721 | 0.9999 |
| 21 | 2.37 | -0.2005 | 0.2149 | 0.8321 | 0.102691 | 0.400821 | 0.3265 | 0.9999 |
| 22 | 2.38 | -0.02213 | 0.8922 | 0.9435 | 0.638986 | 0.783823 | 0.8823 | 0.9999 |
| 23 | 2.42 | -0.1078 | 0.5081 | 0.8975 | 0.116213 | 0.40296 | 0.3527 | 0.9999 |
| 24 | 2.46 | 0.06178 | 0.7049 | 0.9269 | 0.550505 | 0.737966 | 0.7881 | 0.9999 |
| 25 | 2.55 | -0.05236 | 0.7483 | 0.9303 | 0.684552 | 0.817906 | 0.9999 | 0.9999 |
| 26 | 2.64 | -0.04616 | 0.7773 | 0.9409 | 1 | 1 | 0.9999 | 0.9999 |
| 27 | 2.66 | -0.1003 | 0.5379 | 0.8975 | 0.765761 | 0.903205 | 0.9999 | 0.9999 |
| 28 | 2.71 | -0.118 | 0.4685 | 0.8975 | 0.432199 | 0.685557 | 0.9999 | 0.9999 |
| 29 | 2.76 | 0.18 | 0.2663 | 0.8321 | 0.058981 | 0.365359 | 0.1545 | 0.9999 |
| 30 | 2.81 | -0.05345 | 0.7432 | 0.9303 | 0.870909 | 0.977259 | 0.9999 | 0.9999 |
| 31 | 2.87 | 0.0261 | 0.873 | 0.9416 | 0.371445 | 0.683459 | 0.9999 | 0.9999 |
| 32 | 2.91 | 0.01155 | 0.9436 | 0.9819 | 0.329549 | 0.683459 | 0.9999 | 0.9999 |
| 33 | 2.95 | 0.2985 | 0.0613 | 0.5727 | **0.037947** | 0.323725 | 0.1665 | 0.9999 |
| 34 | 2.99 | 0.1063 | 0.514 | 0.8975 | 0.498343 | 0.729851 | 0.9999 | 0.9999 |
| 35 | 3.02 | 0.0246 | 0.8802 | 0.9416 | 0.978393 | 1 | 0.9999 | 0.9999 |
| 36 | 3.05 | -0.1589 | 0.3274 | 0.8321 | **0.014602** | 0.323725 | **0.0147** | 0.9999 |
| 37 | 3.09 | -0.09452 | 0.5618 | 0.8975 | 0.464616 | 0.724486 | 0.9969 | 0.9999 |
| 38 | 3.14 | -0.3335 | **0.0355** | 0.5727 | 0.139817 | 0.440358 | 0.1784 | 0.9999 |
| 39 | 3.19 | -0.1024 | 0.5296 | 0.8975 | 0.371445 | 0.683459 | 0.9999 | 0.9999 |
| 40 | 3.22 | -0.07768 | 0.6338 | 0.9255 | 0.935242 | 0.977753 | 0.9999 | 0.9999 |
| 41 | 3.26 | 0.1145 | 0.4817 | 0.8975 | 0.348157 | 0.683459 | 0.5913 | 0.9999 |
| 42 | 3.29 | -0.1161 | 0.4755 | 0.8975 | 0.331027 | 0.683459 | 0.5282 | 0.9999 |
| 43 | 3.34 | -0.1537 | 0.3437 | 0.8321 | 0.551277 | 0.737966 | 0.9999 | 0.9999 |
| 44 | 3.42 | 0.1653 | 0.3081 | 0.8321 | 0.086374 | 0.39732 | 0.1763 | 0.9999 |
| 45 | 3.48 | -0.06542 | 0.6884 | 0.9269 | 0.371445 | 0.683459 | 0.9245 | 0.9999 |
| 46 | 3.53 | 0.08266 | 0.6121 | 0.9178 | 0.499789 | 0.729851 | 0.8126 | 0.9999 |
| 47 | 3.56 | -0.1862 | 0.25 | 0.8321 | 0.266719 | 0.632854 | 0.842 | 0.9999 |
| 48 | 3.57 | -0.2998 | 0.0602 | 0.5727 | **0.032384** | 0.323725 | 0.1423 | 0.9999 |
| 49 | 3.60 | 0.09133 | 0.5752 | 0.8975 | 0.268275 | 0.632854 | 0.5269 | 0.9999 |
| 50 | 3.69 | -0.1543 | 0.3417 | 0.8321 | 0.416494 | 0.685557 | 0.9999 | 0.9999 |
| 51 | 3.71 | -0.05859 | 0.7195 | 0.9269 | **0.039554** | 0.323725 | 0.2331 | 0.9999 |
| 52 | 3.73 | 0.001816 | 0.9911 | 0.9977 | 0.892275 | 0.977259 | 0.9999 | 0.9999 |
| 53 | 3.76 | 0.1982 | 0.2201 | 0.8321 | **0.027244** | 0.323725 | 0.1087 | 0.9999 |
| 54 | 3.81 | -0.349 | **0.0273** | 0.5727 | 0.07833 | 0.39732 | 0.1712 | 0.9999 |
| 55 | 3.85 | 0.03885 | 0.8119 | 0.9416 | 0.935242 | 0.977753 | 0.9999 | 0.9999 |
| 56 | 3.89 | -0.03554 | 0.8276 | 0.9416 | 0.371445 | 0.683459 | 0.9999 | 0.9999 |
| 57 | 3.93 | 0.04262 | 0.794 | 0.9416 | 0.569517 | 0.737966 | 0.9999 | 0.9999 |
| 58 | 3.98 | 0.2576 | 0.1086 | 0.5877 | **0.042225** | 0.323725 | 0.0986 | 0.9999 |
| 59 | 4.06 | 0.09229 | 0.5711 | 0.8975 | 0.367787 | 0.683459 | 0.6579 | 0.9999 |
| 60 | 4.12 | -0.3066 | 0.0543 | 0.5727 | 0.058883 | 0.365359 | 0.1731 | 0.9999 |
| 61 | 4.18 | -0.1749 | 0.2805 | 0.8321 | 0.849493 | 0.977259 | 0.9999 | 0.9999 |
| 62 | 4.21 | 0.3242 | **0.0413** | 0.5727 | 0.227894 | 0.613459 | 0.7826 | 0.9999 |
| 63 | 4.26 | -0.1121 | 0.4911 | 0.8975 | 0.606836 | 0.76478 | 0.9999 | 0.9999 |
| 64 | 4.32 | -0.2696 | 0.0925 | 0.5727 | 0.175673 | 0.50506 | 0.8637 | 0.9999 |
| 65 | 4.41 | 0.1224 | 0.4519 | 0.8975 | 0.53333 | 0.737966 | 0.9999 | 0.9999 |
| 66 | 4.44 | 0.1714 | 0.2904 | 0.8321 | 0.093112 | 0.400821 | 0.2853 | 0.9999 |
| 67 | 4.52 | 0.1051 | 0.5188 | 0.8975 | 0.329549 | 0.683459 | 0.9803 | 0.9999 |
| 68 | 4.58 | 0.343 | **0.0302** | 0.5727 | **0.013482** | 0.323725 | 0.0629 | 0.9999 |
| 69 | 4.65 | 0.05736 | 0.7252 | 0.9269 | 0.569517 | 0.737966 | 0.9999 | 0.9999 |
| 70 | 5.88 | 0.2654 | 0.0979 | 0.5727 | 0.143595 | 0.440358 | 0.4133 | 0.9999 |
| 71 | 5.92 | 0.3108 | 0.0509 | 0.5727 | **0.014787** | 0.323725 | **0.0393** | 0.9999 |
| 72 | 5.97 | 0.1135 | 0.4856 | 0.8975 | 0.935242 | 0.977753 | 0.9999 | 0.9999 |
| 73 | 6.09 | 0.2829 | 0.0769 | 0.5727 | **0.016914** | 0.323725 | **0.0359** | 0.9999 |
| 74 | 6.52 | 0.1195 | 0.4628 | 0.8975 | 0.588039 | 0.751383 | 0.9999 | 0.9999 |
| 75 | 6.61 | 0.0479 | 0.7691 | 0.9409 | 0.551277 | 0.737966 | 0.9999 | 0.9999 |
| 76 | 6.79 | -0.2249 | 0.163 | 0.7893 | 0.110053 | 0.40296 | 0.3122 | 0.9999 |
| 77 | 6.88 | 0.000474 | 0.9977 | 0.9977 | 0.343161 | 0.683459 | 0.9517 | 0.9999 |
| 78 | 6.99 | -0.06188 | 0.7045 | 0.9269 | 0.989134 | 1 | 0.9999 | 0.9999 |
| 79 | 7.17 | 0.0696 | 0.6696 | 0.9269 | 0.244177 | 0.624008 | 0.7708 | 0.9999 |
| 80 | 7.20 | 0.03191 | 0.845 | 0.9416 | 0.401131 | 0.685557 | 0.9999 | 0.9999 |
| 81 | 7.31 | -0.1833 | 0.2576 | 0.8321 | 0.684552 | 0.817906 | 0.9999 | 0.9999 |
| 82 | 7.36 | 0.1582 | 0.3297 | 0.8321 | 0.416494 | 0.685557 | 0.9999 | 0.9999 |
| 83 | 7.41 | -0.05731 | 0.7254 | 0.9269 | 0.956801 | 0.989053 | 0.9999 | 0.9999 |
| 84 | 7.73 | -0.1066 | 0.5125 | 0.8975 | 0.922746 | 0.977753 | 0.9999 | 0.9999 |
| 85 | 7.90 | 0.2453 | 0.1272 | 0.6501 | 0.083029 | 0.39732 | 0.2773 | 0.9999 |
| 86 | 7.96 | -0.01025 | 0.9499 | 0.9819 | 0.432199 | 0.685557 | 0.9999 | 0.9999 |
| 87 | 8.17 | -0.2758 | 0.085 | 0.5727 | 0.104154 | 0.400821 | 0.4682 | 0.9999 |
| 88 | 8.23 | 0.2677 | 0.0949 | 0.5727 | **0.039554** | 0.323725 | 0.1711 | 0.9999 |
| 89 | 8.35 | 0.2641 | 0.0996 | 0.5727 | **0.019847** | 0.323725 | 0.0938 | 0.9999 |
| 90 | 8.41 | 0.08356 | 0.6082 | 0.9178 | 0.631989 | 0.783823 | 0.9107 | 0.9999 |
| 91 | 8.60 | -0.1941 | 0.23 | 0.8321 | 0.155424 | 0.461258 | 0.2797 | 0.9999 |
| 92 | 8.93 | 0.1351 | 0.4057 | 0.8975 | 0.498343 | 0.729851 | 0.9999 | 0.9999 |

q-value is based on Benjamini-Hochberg correction.
